# Transmission-Blocking Strategies for Malaria Eradication: Recent Advances in Small-Molecule Drug Development

**DOI:** 10.3390/ph17070962

**Published:** 2024-07-19

**Authors:** Federico Appetecchia, Emanuele Fabbrizi, Francesco Fiorentino, Sara Consalvi, Mariangela Biava, Giovanna Poce, Dante Rotili

**Affiliations:** Department of Drug Chemistry and Technologies, Sapienza University of Rome, P.le A. Moro 5, 00185 Rome, Italy; federico.appetecchia@uniroma1.it (F.A.); emanuele.fabbrizi@uniroma1.it (E.F.); f.fiorentino@uniroma1.it (F.F.); sara.consalvi@uniroma1.it (S.C.); mariangela.biava@uniroma1.it (M.B.)

**Keywords:** malaria, transmission blocking, plasmodium, mosquito, gametocytes, oocyst

## Abstract

Malaria drug research and development efforts have resurged in the last decade following the decelerating rate of mortality and malaria cases in endemic regions. The inefficiency of malaria interventions is largely driven by the spreading resistance of the *Plasmodium falciparum* parasite to current drug regimens and that of the malaria vector, the *Anopheles* mosquito, to insecticides. In response to the new eradication agenda, drugs that act by breaking the malaria transmission cycle (transmission-blocking drugs), which has been recognized as an important and additional target for intervention, are being developed. These drugs take advantage of the susceptibility of *Plasmodium* during population bottlenecks before transmission (gametocytes) and in the mosquito vector (gametes, zygotes, ookinetes, oocysts, sporozoites). To date, compounds targeting stage V gametocytes predominate in the chemical library of transmission-blocking drugs, and some of them have entered clinical trials. The targeting of *Plasmodium* mosquito stages has recently renewed interest in the development of innovative malaria control tools, which hold promise for the application of compounds effective at these stages. In this review, we highlight the major achievements and provide an update on the research of transmission-blocking drugs, with a particular focus on their chemical scaffolds, antiplasmodial activity, and transmission-blocking potential.

## 1. Introduction

Despite substantial progress in the field, malaria remains a global health issue, accounting for more than 250 million cases and more than 600,000 deaths in 2021, especially among young children and in Sub-Saharan Africa, and is one of the deadliest infectious diseases in the world [1]. Cases are predicted to increase due to evolving resistance to the current malaria control and elimination strategies as a consequence of mutations in the drug target proteins. Additionally, the recent coronavirus disease 2019 (COVID-19) may pose a threat to malaria control in several ways. Indeed, access to health care was restricted during the pandemic, and malaria interventions, such as the distribution of long-lasting insecticide-treated bed nets and seasonal malaria chemoprevention, were suspended due to lockdowns [2]. Therefore, there is an urgent need to tackle the spread of this disease with novel therapies that can be added to existing interventions to reach the targets of the Global Technical Strategy, which aims to reduce malaria mortality and case incidence by at least 90% by 2030 [1].

**Figure 1:**
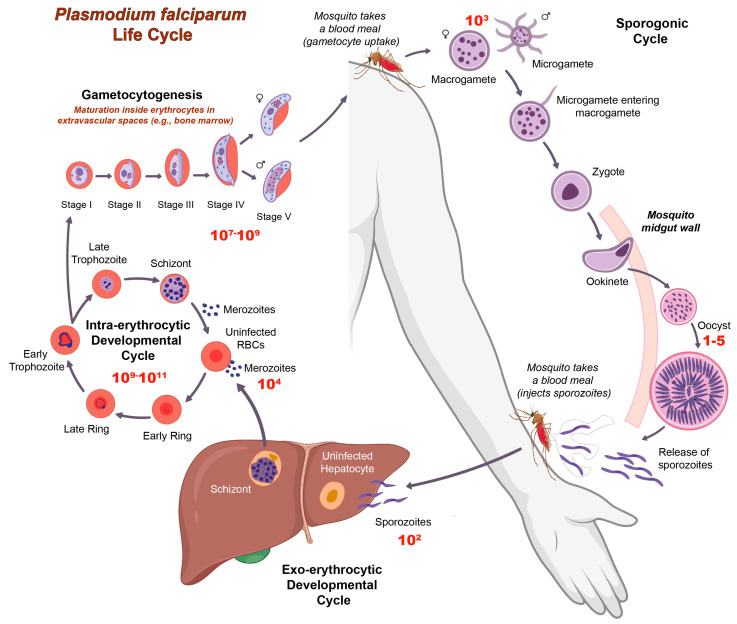
The malaria parasite life cycle. *Plasmodium* sporozoites are injected into the human host’s dermis during an *Anopheles* mosquito blood meal before making their way to the liver. Hepatic schizogony begins when a sporozoite invades a hepatocyte, and the resultant merozoites (10^4^) enter the bloodstream to start the symptomatic ABS characterized by the presence of 10^9^–10^11^ parasites in total. A small percentage of asexual parasites engage in gametocytogenesis, producing adult male and female gametocytes (10^7^–10^9^ in total) in a development process that lasts 10–12 days. Roughly 103 gametocytes are transmitted to *Anopheles* mosquitoes following a blood meal. The midgut of the mosquito activates gametogenesis, which is followed by fertilization to form a diploid zygote, which, during meiosis, elongates into a tetraploid ookinete within ~24 h. Ookinetes develop in six morphologically distinct stages and progress to oocysts (~48 h) by penetrating the midgut wall. Each oocyst (1–5 in total) is attached to the basal lamina of the midgut and replicates its genome for the next 6–12 days to develop hundreds of sporozoites inside the cellular membrane (sporogony). The cycle is restarted when sporozoites develop and migrate to the salivary glands of the mosquito to infect another human host. Created with Biorender.com.

In response to the increasing drug resistance of asexual blood-stage (ABS) *Plasmodium* spp. to common antimalarial drugs, scientists are studying new methodologies that aim to stop the spread of malaria by targeting the bottlenecks of the *Plasmodium* life cycle (Figure 1) [3,4,5,6]. Of 1000 mature gametocytes consumed by a female mosquito *Anopheles* during a blood meal, only 50 to 100 of them (~1% of gametocytes) are thought to escape immune factors and mature into ookinetes, which leads to fewer than 5 parasites per mosquito in the oocyst stage (Figure 1). Moreover, a significant number of sporozoites are lost on the way from the skin to the human liver [7,8]. Therefore, targeting gametocytes and other parasitic stages/events that occur in mosquitoes (gamete formation, fecundation, zygote formation, and ookinete maturation) with the use of transmission-blocking tools would prevent a mosquito from infecting humans.

Transmission-blocking strategies can either target the mosquito vector (insecticides or endectocides) or the parasite life cycle. Methods based on the latter include transmission-blocking vaccines, transmission-blocking endosymbionts (e.g., *Wolbachia*), and transmission-blocking drugs [9,10,11]. While the first two subcategories are more difficult to implement in the field and require long processes, the development of transmission-blocking drugs, also known as transmission blockers, seems more feasible at the moment. This approach bypasses the ethical and technical issues of the other two strategies, such as those related to the spread of *Wolbachia*-infected mosquitoes, which require in-depth ecological studies [10,11].

By using transmission blockers, the chance of emerging resistance would be largely decreased. Moreover, they could also synergize with anti-asexual agents to prevent the escape of resistant mutants and slow down the evolution of drug-resistant parasites [12,13]. To date, primaquine is the only medicine approved by the World Health Organization (WHO) for transmission-blocking purposes, and it can be used in conjunction with artemisinin in low- to moderate-transmission settings [13]. Nevertheless, this strategy is not extensively utilized because of the toxicity issues emerging in glucose-6-phosphate dehydrogenase (G6PD)-deficient individuals [14].

The most common targets for transmission-blocking drugs are late-stage gametocytes (IV-V), which seem more amenable to therapeutic intervention, as they can be easily targeted within the human blood compartment [15]. Unlike anti-asexual drugs, these transmission-blocking agents target not only gametocytes in symptomatic carriers but also those in asymptomatic individuals, who may be responsible for up to 84% of persistent malaria transmission. Therefore, the widespread administration of drugs, regardless of symptoms, would be required, and this strategy will need to face ethical and compliance hurdles before being implemented in the field [13]. 

Yet, there is a significant population bottleneck in the vector midgut, highlighting that sporogonic stages in mosquitoes (gametes, zygotes, ookinetes, and oocysts) are valuable targets for innovative strategies to block transmission [7,16]. The development of drugs targeting sporogonic stages has been hampered by the insufficiency of knowledge about their biology and technical obstacles. *P. falciparum* mature gametocytes can circulate in human blood for up to three weeks [17]. Therefore, medications need to possess prolonged half-lives to efficiently disrupt the vital functions of all circulating gametocytes and target the sporogonic stages following blood absorption. In addition to the lack of standardized methods to study the pharmacokinetics (PK) and pharmacodynamics (PD) of drugs inside mosquitoes, there are no in vitro screening methods targeting *P. falciparum* post-gamete formation stages. The only assays currently available for determining the activity of potential drugs against these stages were developed using the rodent malaria parasite *P. berghei* [15,18,19]. 

The discovery by Paton et al. [20] has recently laid the foundation for a novel malaria control strategy based on incorporating antimalarial drugs into mosquito-targeting interventions and has demonstrated that the sporogonic stages of *P. falciparum* parasites can be completely abrogated when *Anopheles gambiae* females are exposed tarsally to surfaces coated with low concentrations of atovaquone. This method sheds new light on the search for new antimalarial compounds active against sporogonic stages, which has been hindered by a lack of knowledge of parasite–vector interaction and a scarcity of testing tools [21]. We believe that with a more complete understanding of post-transmission biology, this strategy, along with the current frontline interventions, has the potential to have a huge epidemiological impact. 

The quest for new transmission-blocking drugs has dramatically increased over the past ten years, resulting in the development of innovative, promising candidates and cutting-edge strategies that have been extensively reviewed [4,5,17,22,23,24,25]. In this review, following a quick overview of the most popular assays used to evaluate potential transmission-blocking drugs, we provide a detailed description of the most relevant transmission-blocking compounds developed between 2020 and 2023. To serve as a reference for the future development of transmission-blocking medicines, we present the chemical structures and bioactivities of the reviewed inhibitors in both the asexual and transmission stages. We also describe the structure–activity relationships (SARs) of the examined compounds, where available.

## 2. The Transmission-Blocking Screening Landscape

Most screening methods for new antimalarial compounds are fluorescence-based assays that measure cell proliferation with a focus on blood-stage parasites cultured in vitro and have been used for a long time as a primary filter to identify and prioritize novel hits [26,27,28]. Since transmission-blocking drugs have become a priority area of investigation, a race has begun to develop effective and rapid screening methods against the other phases of the parasite life cycle to use in parallel with asexual in vitro screening. Such improvement would remove the selection bias associated with ABS-based screening and thus cover a broader chemical space for the identification of novel hits with transmission-blocking potential [25,29,30]. Today, the search for compounds with transmission-blocking activity prioritizes drugs able to prevent infections by blocking parasite transmission from infected individuals to mosquito vectors, since they are more amenable to medium- or high-throughput screening. Indeed, no standardized in vitro assays evaluating drug activity against the *P. falciparum* sporogonic cycle have yet been reported, and the only assays available for investigating compound activity against these stages have been developed for the rodent malaria parasite *P. berghei* [19]. 

### 2.1. Gametocytocidal Assays

**Figure 2:**
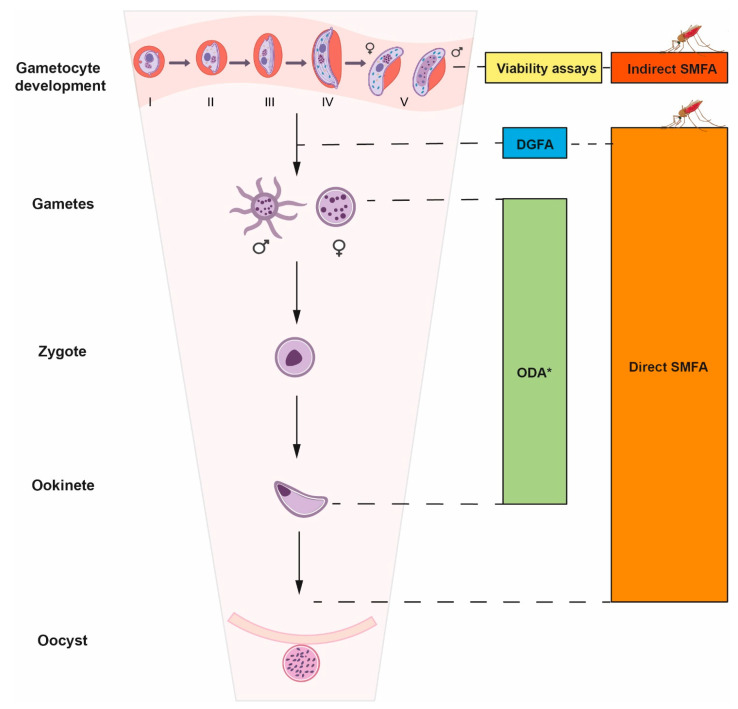
An overview of the most common transmission-blocking assays showing their targets in the *Plasmodium* life cycle. Viability assays are employed to assess the influence of potential drugs on gametocyte development. The DGFA is used to evaluate the ability of compounds to inhibit the production of gametes. The SMFA is employed to assess the transmission-blocking potential of drug candidates. In its indirect form, the SMFA informs on the effect of small molecules on *Plasmodium* gametocytogenesis, whereas in its direct version, it informs on the impact on gamete development into the oocyst. The ODA (* performed in *P. berghei*) enables the assessment of the effects of potential drugs on the early sporogonic development of parasites in the mosquito midgut. DGFA—dual gamete formation assay; ODA—ookinete development assay; SMFA—standard membrane feeding assay. Created with Biorender.com.

Gametocytocidal assays (Figure 2) are usually performed in the widely used laboratory strain *P. falciparum* NF54 (a clone of 3D7, which originates from an African isolate) [31] for both immature and mature gametocyte stages, as it generates the maximum gametocytemia and has good repeatability [32]. Since gametocytes are non-replicative embryonic stages, gametocytocidal activity is assessed by a range of assays that use colorimetric readouts or reporter lines as indicators of metabolic activity (viability marker) [30,33]. Colorimetric assays include the use of indicator dyes sensitive to oxidation–reduction (Alamar Blue and Presto Blue assays) due to parasite lactate dehydrogenase (*Pf*LDH) levels, a crucial enzyme in anaerobic carbohydrate metabolism necessary for ATP synthesis for *Plasmodium* parasites [29,34]. These methods are cheaper, faster, and easier to perform, but unspecific interactions and challenges with the purity of gametocyte culture may decrease the signal-to-noise ratio and interfere with the analysis [34,35]. More expensive alternatives rely on the measurement of a bioluminescence or fluorescence signal that is proportional to gametocyte metabolic activity. These include gametocytes stained with a fluorescent dye (e.g., MitoTracker Red), reagents measuring ATP levels (e.g., BacTiter-Glo), or transgenic parasite lines expressing a specific fluorescent reporter gene, such as the green fluorescent protein (GFP) or GFP-luciferase [34]. 

It is worth mentioning two recent large-scale screening methods: the Saponin-lysis Sexual Stage Assay (SaLSSA) developed by the University of California San Diego School of Medicine and the use of acridine orange to measure gametocytemia and rounding-up post-activation as a marker of viability developed by researchers at the Istituto Superiore di Sanità in Rome [36,37]. All of these assays offer high sensitivity and can be adapted for medium- or high-throughput assays. Nevertheless, result variability is quite common. It is thus recommended to perform more than one method to better assess a compound’s gametocytocidal activity [29,35]. Moreover, they do not reveal the actual ability of gametocytes to infect a mosquito, which is evaluated in the membrane feeding assays described below.

### 2.2. Dual Gamete Formation Assay (DGFA)

To become infected, a mosquito must ingest at least one mature gametocyte from each sex [16]. The investigation of drug selectivity toward male or female gametes in gametocytocidal tests was not possible before the development of modern gametocytocidal assays, which allowed an improvement in the transmission-blocking efficacy of newly developed compounds. Gametocytocidal assays evaluate the transmission-blocking activity of compounds based on their ability to inhibit the production of gametes (gametogenesis) by mature female and/or male gametocytes. Among these, the DGFA (Figure 2) has been the most effective and has therefore recently been adapted to the 384-well format [38]. In this assay, mature gametocytes are exposed to test compounds, and gametogenesis is induced after a given incubation period and under the appropriate conditions. The development of “exflagellation centers” is an indicator of male gamete production, whereas immunostaining of a surface protein expressed at the gamete surface upon egress allows the detection of female gamete production [39].

Strong evidence of a linear relationship between the DGFA and the standard membrane feeding assay (SMFA) has proven to be an effective high-throughput indicator of the transmission-blocking potential of tested compounds [38,40]. 

### 2.3. SMFA

To date, all hits from transmission-blocking screening need to be validated through the SMFA (Figure 2), which is considered the gold-standard assay to evaluate transmission-blocking activity [41,42]. In its “indirect” form, this test typically entails the infection of *Anopheles* mosquitoes using an artificial membrane to cover a vessel containing blood infected with mature gametocytes previously exposed to transmission-blocking candidates. The parasite viability is then evaluated 7–10 days after the infected blood meal by counting the number of oocysts developed in the mosquito midgut [41,43]. 

Variations of this assay include the “indirect washout” SMFA, in which the candidate compounds are washed out of the infectious blood meal prior to blood feeding, and the “direct” SMFA, in which gametocytes are exposed to candidate compounds immediately before mosquito blood feeding. These variants of the SMFA are utilized to provide additional information regarding the selectivity of the tested hits [35]. The major drawback of the SMFA is its limited throughput. Researchers are thus working to enhance transmission-blocking assays that can predict SMFA outcomes. Among these, the DGFA shows promise as a future gold standard for the transmission-blocking activity of compounds targeting gametocytes; however, additional research is required to validate its efficacy [25,39]. 

### 2.4. Sporogonic Development Assays Using P. berghei

The ookinete development assay (ODA), initially described by Delves et al. [19], allows the evaluation of the effect of drug candidates on the early sporogonic development (between gametogenesis and ookinete maturation) of parasites in mosquitoes (Figure 2). In this assay, GFP-expressing *P. berghei* gametocytes from an infected mouse are exposed to the compound and simultaneously induced to form gametes in a medium simulating the mosquito midgut. After 22–24 h, when mature ookinetes should have formed, high-content imaging microscopy is used to determine the compound-induced mortality rate by counting GFP-expressing parasites.

More recently, Azevedo et al. [18,44] reported a luminescence-based assay using *P. berghei* to test the activity toward oocyst formation and maturation. Oocyst formation is evaluated by treating purified ookinetes with test compounds for 72 h, while the evaluation of oocyst development inhibition is performed after the incubation of the early oocyst with the target molecule for 12 days. Even though these screening methods can be performed in a 384-well format, they do not detect compounds with *P. falciparum*-specific activity, and adapting them to *P. falciparum* remains a top priority.

## 3. Transmission-Blocking Antimalarial Drugs

### 3.1. Transmission Blockers in Clinical Development

As a preface to the description of the different transmission-blocking compounds reported between 2020 and 2023, here, we summarize the properties of currently available transmission-blocking antimalarial drugs currently under study in clinical trials [4].

**Figure 3:**
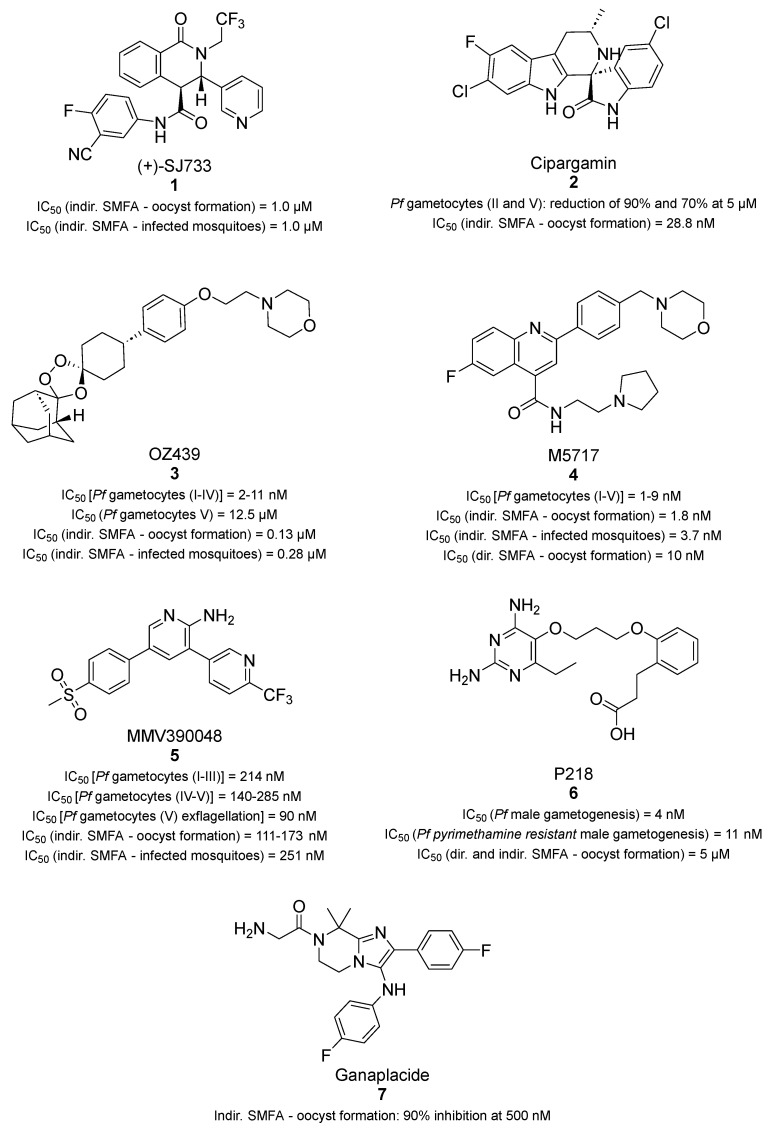
Structures and transmission-blocking activities of compounds **1**–**7** currently in clinical phases.

The tetrahydroisoquinoline (+)-SJ733 (**1**, Figure 3) showed good antiplasmodial activity with potent in vitro activity against the ABS of different strains of *P. falciparum*, with IC_50_ values ranging from 10 to 60 nM, and was equally potent against all asexual stages of the erythrocytic life cycle. Compound **1** was also efficacious in vivo with a 90% effective dose (ED_90_) of 1.9 mg/kg and blocked the transmission of *P. berghei* from infected mice to mosquitoes with an ED_50_ value of 5 mg/kg [45]. Mechanistically, **1** was indicated to target the *P. falciparum* Na^+^-efflux ATPase ATP4 (*Pf*ATP4), thereby increasing the intracellular Na^+^ concentration ([Na^+^]_i_), with an IC_50_ value of 200 nM. In another study, Dechering et al. performed an indirect SMFA by incubating compound **1** with *P. falciparum* NF54 stage V gametocytes for 24 h at 37 °C before mosquito feeding. Compound **1** reduced both oocyst formation and the normalized prevalence of infected mosquitoes with IC_50_ values of 1.0 and 1.6 µM [46]. Given its optimal pharmacokinetics (PK), safety profile, and in vivo activity, compound **1** has recently been tested in human subjects in a Phase 1 clinical trial (NCT02867059) [47,48]. Compound **1** exhibited fast-acting behavior with a favorable drug-likeness profile when tested in direct skin feeding or indirect membrane feeding of mosquitoes on human patients with infections (>500 parasites/mL), although its quick metabolism prevents a single-dose treatment. Interestingly, Gaur et al. demonstrated that the association of **1** with the potent CYP3A4 inhibitor cobicistat improved drug exposure and the PK parameters of compound **1**, allowing for a single-dose administration [48]. 

Cipargamin (**2**, Figure 3), a spiroindolone-based inhibitor of *Pf*ATP4, displayed potent dose-dependent inhibition of *P. vivax* and *P. falciparum* (including in multidrug-resistant parasites) ABSs with IC_50_ values of 0.5–1.4 nM [49]. Furthermore, it showed activity against all stages of gametocyte and sporogonic development of *P. falciparum* in vitro. Indeed, compound **2** reduced stage II and stage V gametocyte counts in red blood cells (RBCs) by ~90% and ~70% at 5 nM, respectively, while abolishing 100% gametocyte development at both 50 and 500 nM [50]. In addition, a clear damaging effect on stage II gametocyte morphology was observed, with the remaining gametocytes showing swollen, rounded forms. Moreover, **2** reduced oocyst formation dose-dependently (IC_50_ = 28.8 nM) in SMFAs and decreased the prevalence of infected mosquitoes with an IC_50_ of 43.7 nM [46]. Interestingly, a recent Phase 2a clinical trial (NCT03334747) showed that recrudescent parasites bearing a G358S mutation in *Pf*ATP4 presented high levels of resistance to **2** and were found in patients with uncomplicated malaria. This mutation causes a decrease in the affinity of *Pf*ATP4 toward Na^+^ and is linked to an increase in the parasite’s cytosolic [Na^+^] at rest [51,52]. 

OZ439 (also known as artefenomel, **3**, Figure 3) is an endoperoxide-containing compound active against multiple *P. falciparum* stages. Specifically, **3** displayed a low-nanomolar IC_50_ against gametocyte stages I–IV (IC_50_ values of 11, 5, 3, and 2 nM, respectively) while not being active against stage V gametocytes (IC_50_ > 12.5 µM) [36]. In an indirect SMFA, **3** could eliminate 100% of oocysts at 1 µM [36]. In line with this, later studies that performed SMFAs at multiple doses of **3** indicated a decrease in oocyst formation with an IC_50_ value of 0.13 µM and a reduction in the prevalence of infected mosquitoes with an IC_50_ of 0.28 µM [46]. Nonetheless, its activity against the ABS was shown to be almost 2 orders of magnitude higher (IC_50_ = 1.9 nM) [53]. Although the target of **3** has not been disclosed yet, the most recognized theory involves the perturbation of heme metabolism and hemoglobin digestion. Interestingly, the results mentioned above correlate well with previous studies reporting that hemoglobin digestion ends at stages III to IV, supporting the notion that **3** may target this pathway [54,55,56]. 

M5717 (also known as DDD107498, **4**, Figure 3) is a quinoline-4-carboxamide that inhibits *P. falciparum* translation elongation factor 2 (*Pf*eEF2), a protein involved in the promotion of the GTP-dependent translocation of the ribosome along messenger RNA during protein synthesis [57]. In line with this, **4** specifically inhibits *P. falciparum* 3D7 protein synthesis (IC_50_ = 2 nM), which is crucial in all *Plasmodium* life-cycle stages, thereby explaining the significant multistage activity of **4**. It showed excellent activity against 3D7 asexual parasites with an IC_50_ value of 1.0 nM, and it was efficacious in a *P. berghei*-infected mouse model (ED_90_ = 0.57 mg/kg) after a single oral dose. Compound **4** strongly inhibited *P. falciparum* male and female gamete formation at similar concentrations (1.8 nM and 1.2 nM, respectively) and was active against all gametocyte stages in the low-nanomolar range (IC_50_ values for stages I-V of 3, 5, 5, 1, and 9 nM, respectively [36]). In an indirect SMFA, **4** was incubated with *P. falciparum* stage V gametocytes for 24 h before mosquito feeding and blocked oocyst development in the mosquito with an IC_50_ of 1.8 nM and decreased the prevalence of infected mosquitoes with an IC_50_ value of 3.7 nM. Moreover, a direct SMFA showed that **4** could impair oocyst development in the mosquito midgut with an IC_50_ value of 10 nM [57]. In addition, **4** prevented mouse-to-mouse *P. berghei* transmission. Indeed, an oral dose of 3 mg/kg of compound **4** administered 24 h before mosquitoes had their blood meal resulted in a 90.7% reduction in infected mosquitoes and a 98.8% reduction in oocysts per midgut at day 10. Moreover, the authors observed an 89.5% reduction in the number of drug-treated mosquito-bitten mice developing blood-stage infections as compared with non-treated mosquito-bitten mice [58].

The 2-aminopyridine MMV390048 (**5**, Figure 3) was obtained from a high-throughput phenotypic screening and showed promising results as a multistage antimalarial compound. In addition to its activity as a nanomolar inhibitor of ABS parasites and liver-stage parasites, **5** was also active as a transmission-blocking agent, albeit to a lesser extent [59]. Compound **5** inhibited the viability of stage I–III gametocytes with an IC_50_ value of 214 nM and had a similar potency against stage IV–V gametocytes (IC_50_ values of 285 nM and 140 nM in luciferase-expressing gametocytes). Kinetic experiments indicated that luciferase-expressing late-stage gametocytes of five different clinical isolates of *P. falciparum* were killed 2.5 times faster than early-stage gametocytes [59]. Compound **5** also inhibited the exflagellation of stage V gametocytes with an IC_50_ value of 90 nM. An indirect SMFA indicated that the formation of oocysts in the mosquito midgut was inhibited with an IC_50_ value of 111 nM (or 173 nM, depending on the study [46]), while the prevalence of infected mosquitoes was reduced with an IC_50_ of 251 nM [46]. Compound **5**, on the other hand, inhibited the development of oocysts by less than 25% in a direct SMFA at 1 µM. This indicates that it is more potent against stage V gametocytes in the host blood than later forms maturing in the mosquito midgut [59]. The oral administration of **5** at 2 mg/kg to *P. berghei-*infected mice reduced oocyst formation in the mosquito vector by 69.3% and the number of infected mosquitoes (i.e., the prevalence) by 30.3%. Moreover, **5** decreased sporozoite formation by 37.2% and sporozoite prevalence by 46.5%, while it reduced the number of drug-treated mice developing blood-stage infections as compared with non-treated mosquito-bitten mice by only 10.1% [59]. Chemoproteomics identified *P. falciparum* phosphatidylinositol 4-kinase type III β (*Pf*PI4Kβ) as the target of MMV390048, and an enzyme assay on recombinant *P. vivax* PI4K resulted in potent inhibition, with an IC_50_ value of 3.4 nM [59].

In 2012, Yuthavong et al., in partnership with the Medicines for Malaria Venture (MMV) initiative, developed the potent *P. falciparum* dihydrofolate reductase (*Pf*DHFR) inhibitor P218 (**6**, Figure 3) through a structure-based approach [60]. Compound **6** is a hybrid molecule combining the pyrimidine moiety of the antiparasitic agent pyrimethamine with a flexible side chain. These features allowed the drug resistance of quadruple pyrimethamine-resistant mutant *P. falciparum* (V1/S) to be overcome while maintaining the potent activity and drug-like properties of common anti-folate drugs. The flexible 2′-carboxyethylphenyl group chain extends the target residence duration by tightly binding to both wild-type and mutant *Pf*DHFR in a slow-on/slow-off manner. Compound **6** was shown to possess transmission-blocking activity by impairing male gametogenesis, with an IC_50_ value of 4 nM for wild-type *P. falciparum* and 11 nM for the pyrimethamine-resistant mutant. Moreover, it could block oocyst formation in both direct and indirect SMFAs at 5 μM (inhibition > 97%) [61,62]. Compound **6** has entered a Phase 1 clinical trial (NCT02885506) and demonstrated favorable safety, tolerability, and PK profiles along with outstanding chemoprotective activity against *P. falciparum* [22,63,64]. 

Ganaplacide (also known as KAF156, **7**, Figure 3) is an imidazole-pirazine that showed both ABS parasite inhibition and transmission-blocking activity. Compound **7** was found to impair the parasite’s protein secretory pathway, but its specific target is currently under debate [4,65]. Compound **7** has low-nanomolar activity against both asexual blood- and hepatic-stage parasites, which translates into therapeutic and prophylactic activities in mouse models of infection. Furthermore, **7** was shown to inhibit the maturation of stage II gametocytes, with >75% and 100% reductions in stage V gametocytes at 5 and 50 nM, respectively. In an indirect SMFA, treatment with 500 nM KAF156 led to a 90% reduction in oocyst numbers, suggesting that **7** has a profound effect on the final steps of gametocyte maturation. In addition, **7** showed a clear dose-dependent effect, with a 90% reduction in oocyst numbers at a concentration of 500 nM. Moreover, *P. berghei*-infected mice treated with a single oral dose of **7** at 100 mg/kg were found not to be infectious to *Anopheles* mosquitoes feeding on their blood, thereby confirming the transmission-blocking potential [65].

### 3.2. Epigenetic Transmission-Blocking Drugs

The sexual differentiation commitment involves various epigenetic factors, as many genes need to be silenced or expressed to allow gametocyte maturation [66]. Consequently, interfering with these pathways may dramatically alter this process, ultimately preventing parasite transmission.

Vanheer et al. recently evaluated the activity of 350 different epigenetic and kinase inhibitors against multiple stages of *P. falciparum* [67]. Among these compounds, 32 showed an EC_90_ below 1 μM against early gametocyte stages, while only a few compounds showed activity against mature gametocytes at 1 μM, possibly because the epigenetic changes that underlie sexual differentiation are initiated during earlier stages of gametocytogenesis. Despite this, 13 compounds (Table 1) exhibited substantial activity against all three stages (asexual, early gametocyte, and late gametocyte stages) of the *P. falciparum* NF54 strain at 1 μM. These compounds comprise one DNA methyltransferase (DNMT) inhibitor (SGI-1027, **8**), four histone methyltransferase (HMT) inhibitors [chaetocin (**9**), BIX01294 (**10a**), UNC0631 (**10b**), and UNC0642 (**10c**)], one lysine demethylase (KDM) inhibitor (JIB-04, **11**), and seven histone deacetylase (HDAC) inhibitors [quisinostat (**12a**), panobinostat (**13**), apicidin (**14**), HC Toxin (**15**), CUDC-101 (**16**), trichostatin A (**17**), and dacinostat (**18**)]. Moreover, three HMT inhibitors [UNC0679 (**10d**), UNC0638 (**10e**), and UNC0646 (**10f**)] and the kinase inhibitor fedratinib (**19**) possessed IC_50_ values lower than 100 nM against stage I–II gametocytes while not being active against stage IV–V gametocytes [67]. Compound **12a** was the most potent multistage active compound, with IC_50_ values against all three stages in the low-nanomolar range. Most of these compounds displayed great toxicity against human HepG2 cells, with 76–99% inhibition at 1 μM. Some exceptions are **10a** and **19**, which decreased HepG2 viability by ~40% at 1 µM, and all of the other quinazoline-based HMT inhibitors (**10b**–**f**). The latter exhibited promising selectivity, with multistage antiplasmodial activity in the nanomolar range and less than 20% cytotoxicity at 1 μM [67]. In any case, all of the reported compounds represent promising lead molecules for further development, and some of them have been identified in other screens and/or optimized to obtain improved multistage or transmission-blocking antiplasmodial agents, as reported in the following paragraphs.

**Figure 4:**
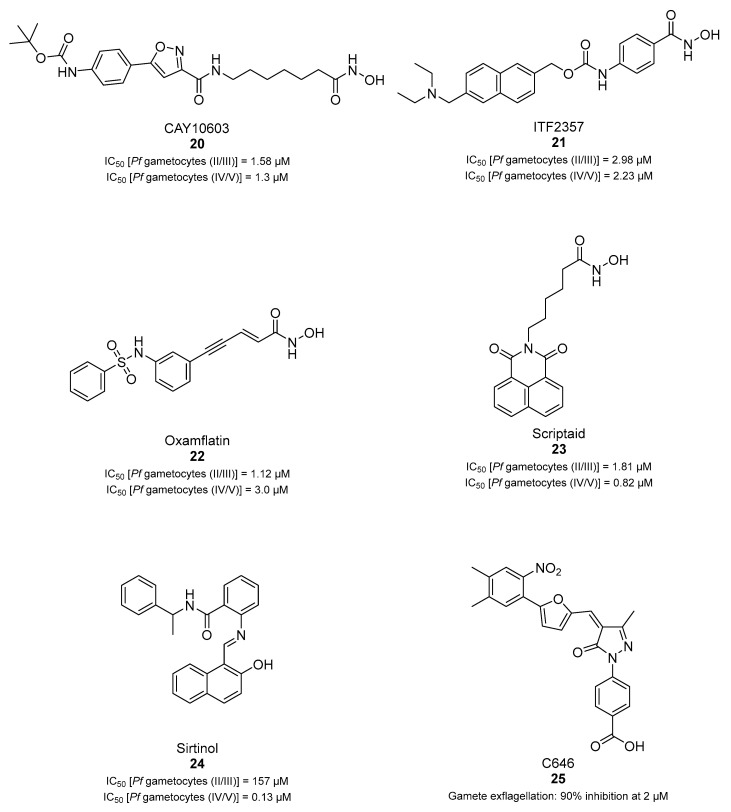
Structures and biological activities of epigenetic inhibitors **20**–**25** identified by Coetzee et al. as transmission-blocking antiplasmodial agents.

A screening of 95 molecules from the Cayman Epigenetics Library led to the identification of 10 compounds active against early- or late-stage gametocytes, or both (Figure 4) [68]. The already-mentioned HMT inhibitors **9**, **10a**, and **10e**, along with the HDAC inhibitor **15** and four more HDAC inhibitors [CAY10603 (**20**), ITF2357 (**21**), oxamflatin (**22**), and scriptaid (**23**), (Figure 4)], exhibited micromolar to submicromolar inhibition of both early- (stages II–III) and late-stage (stages IV–V) *P. falciparum* NF54 gametocyte viability. Moreover, the sirtuin inhibitor sirtinol (**24**, Figure 4) was only active against late-stage gametocytes. A comparison with the reported IC_50_ values against human cell lines indicated that none of the inhibitors was selective for parasites, except for **20**, with a selectivity index >8. Seven compounds (**9**, **10e**, **20**–**24**, and the p300/HDAC inhibitor C646, **18**) inhibited male gamete formation by >60% at 2 µM. Hence, except for **25** (Figure 4), which has only sterilizing effects on mature gametocytes, all of these compounds also possess gametocidal activity. Furthermore, **9**, **20**, and **22** could reduce the normal 3:1 ratio of female/male mature gametocytes to equal proportions after 48 h of treatment with the selected compounds in a functional gamete formation assay. This suggests that they may also target female gamete formation, which could interfere with shared biological functions rather than sex-specific ones. Finally, it is worth noting that **10a**, **10e**, **15**, **20**, and **22** also target asexual stages of *P. falciparum* with IC_50_ values ranging between 0.03 and 3.7 µM.

**Figure 5:**
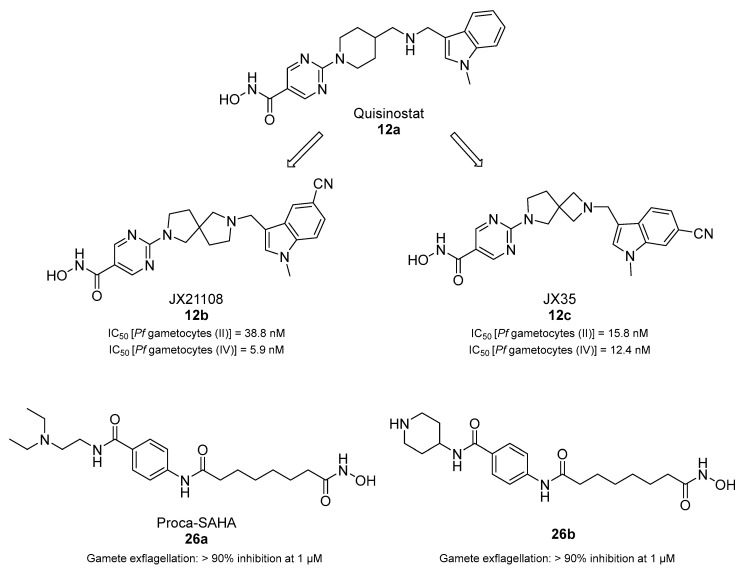
Structures and transmission-blocking activities of compounds **12a**–**c** and **26a**,**b** developed from HDAC inhibitors.

Through a screening by Huang et al., the HDAC inhibitor quisinostat (**12a**, Table 1) was identified as an antiplasmodial agent, showing nanomolar inhibition of ring-stage *P. falciparum* growth and in vivo activity in *P. yoelii*-infected rodents [69]. To improve its toxicity profile, the authors developed new derivatives bearing different linkers connecting the zinc-binding group and the *N*-methylindole moiety. Among them, JX21108 (**12b**, Figure 5), bearing a diazaspiro[4.4]nonane linker and a nitrile function on the indole C5, demonstrated nanomolar activity against different ring-stage *P. falciparum* strains and was less cytotoxic than **12a**. Compound **12b** also showed a 4-fold lower inhibition of human HDACs. Docking and cell-based knockdown studies suggested that its target may be *Pf*HDAC1. Moreover, **12b** displayed potent gametocytocidal activity with IC_50_ values of 38.8 and 5.9 nM against stage II and stage IV *P. falciparum* NF54 gametocytes, respectively. Furthermore, **12b** could stop both liver- and blood-stage *P. berghei* infection at 60 mg/kg. The same research group also developed JX35 (**12c**, Figure 5), a **12a** derivative bearing a 2,6-diazaspiro[3.4]octane linker that has comparable in vitro inhibition, human HDAC selectivity, and cytotoxicity profiles to **12b**, as well as similar activity in vivo [70]. Compound **12c** was also active against stage II and stage IV *P. falciparum* NF54 gametocytes, with IC_50_ values of 15.8 and 12.4 nM, respectively. Both **12a** and **12c** increased *P. falciparum* 3DT histone H3 acetylation and inhibited *Pf*HDAC1 with IC_50_ values of ~0.003 and 0.24 nM, respectively. Finally, knockdown studies confirmed that both **12a** and **12c** target *Pf*HDAC1, in line with the results obtained with **12b**.

Recently, Nardella et al. merged the HDAC inhibitor suberoylanilide hydroxamic acid (SAHA) and the DNMT inhibitor procainamide into one chimeric compound, Proca-SAHA (**26a**), and prepared different derivatives of this compound [71]. Among the tested molecules, **26a** and **26b** (Figure 5), bearing a *N*-piperidin-4-yl in place of a *N*-2-(diethylamino)ethyl moiety, were shown to block both the asexual and transmission stages of the *P. falciparum* life cycle. Both compounds had a similar profile of hHDAC1-3 inhibition compared to SAHA while being more active against hHDAC6 [IC_50_(**26a**) = 14 nM, IC_50_(**26b**) = 19 nM]. Compound **26b** efficiently inhibited histone acetylation in *P. falciparum* extracts (IC_50_ = 480 nM, comparable to **26a**) and increased H4K16 acetylation in *P. falciparum* late-stage cultures 6-fold compared to the DMSO and chloroquine controls. Notably, **26a** was not active against *P. falciparum* nuclear-extract-mediated DNA methylation or human DNMT3A, while **26b** was not assessed. Both compounds impaired the viability of *P. falciparum* NF54 asexual cultures and Cambodian multidrug-resistant isolates in the mid-nanomolar range, with **26b** being less cytotoxic in human HepG2 and HL60 cells than **26a**. Both compounds were tested in stage IIb–III gametocytes for 72 h at 0.5–10 µM and caused abnormal morphology to the same extent as SAHA. Specifically, up to 90% of the gametocytes showed rounded or swollen morphology and presented flagella-like extensions at 10 µM. Moreover, when tested against late-stage gametocytes at the same concentrations, both compounds dramatically reduced the number of exflagellation centers at concentrations starting at 1 µM, while SAHA was active only at 5 µM.

**Figure 6:**
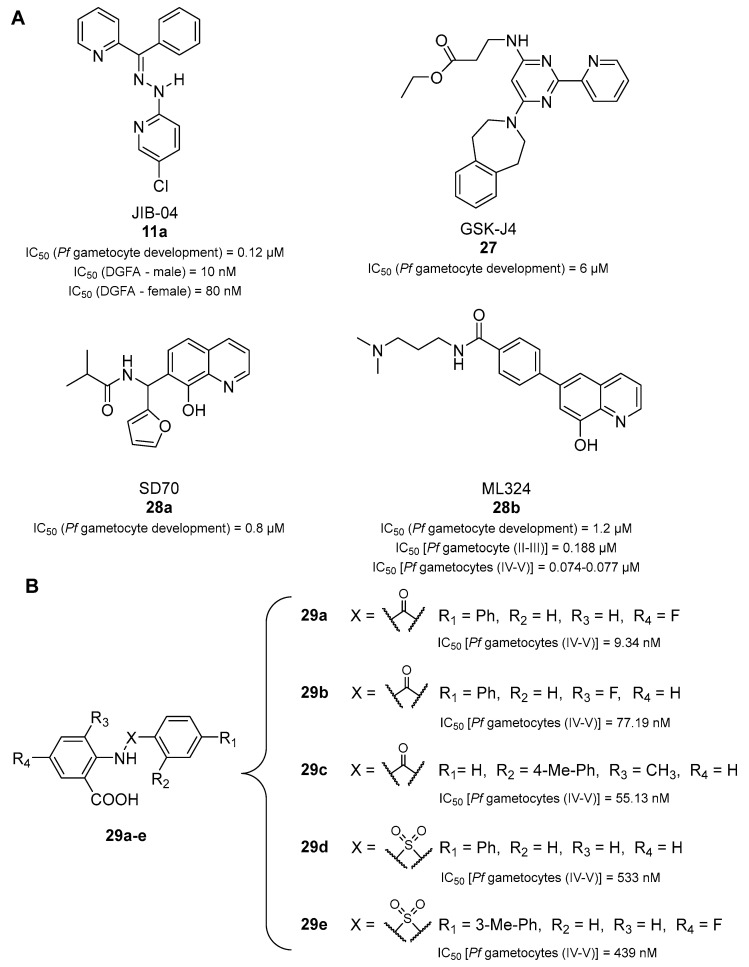
Structures and transmission-blocking activities of KDM inhibitors **11a**, **27**, **28a**,**b** (**A**), and **29a**–**e** (**B**).

Inhibitors of mammalian Jumonji C (JmjC) KDMs have been recently shown to possess antimalarial activity by killing ABS parasites and preventing gametocyte development and gamete formation [72]. The tested compounds were the pan-JmjC inhibitor JIB-04 (**11a**), the KDM6A/B inhibitor GSK-J4 (**27**), and the KDM4 subfamily inhibitors SD-70 (**28a**) and ML324 (**28b**) (Figure 6A). Compounds **11a**, **28a**, and **28b** were shown to inhibit *Pf*Jmj3 with IC_50_ values of 2.6, 2.4, and 3.3 µM, respectively. Mechanistically, these molecules were shown to block the first step of JmJC-catalyzed reactions, namely, the transformation of α-ketoglutarate to succinate. Conversely, **27** displayed an IC_50_ value > 15 µM, although it was active in parasites. Indeed, all compounds impaired the asexual blood-stage parasite growth of both drug-sensitive 3D7 and multidrug-resistant Dd2 *P. falciparum* with IC_50_ values of 1.6–5 μM and delayed the progression of ring- or trophozoite-treated ABS parasites. Compounds **11a**, **28a**, and **28b** were highly effective in preventing gametocyte development, with IC_50_ values of 0.12, 0.8, and 1.2 µM, respectively, while **27** was slightly less potent, with an IC_50_ value of 6 µM. Compound **11a** also impaired gamete formation, as indicated by the inhibition of male exflagellation centers with an IC_50_ of 10 nM and female gamete formation with an IC_50_ of 80 nM. Notably, treatment with **11a** determined an increase in H3K4me3, H3K9me3, and H4K20me3 levels, while treatment with **27** only increased H3K4me3 and H4K20me3 levels, suggesting that these methylation marks may be involved mostly in the sexual stages of the *P. falciparum* life cycle. Finally, RNA sequencing indicated that **11a** downregulated 235 genes and upregulated 385, and gene ontology analysis showed that the alteration of gene expression mostly regarded known invasion and gametocyte transcription- and chromatin-binding factor targets.

In another study, **28b** was found to increase H3K9me3 levels in late-stage gametocytes while not affecting acetylation. Compound **28b** decreased the viability of asexual parasites and early- and late-stage gametocytes, being significantly more potent against late-stage gametocytes compared to ABSs (*p* = 0.0036 ABSs vs. stage IV–V, two-tailed *t* test). Specifically, the IC_50_ values against ABS, stage II–III, and stage IV–V parasites were 2.06, 0.188, and 0.077 µM, respectively [30]. Treatment of *P. falciparum* gametocytes with **28b** caused the downregulation of known H3K9me3-associated genes involved in cell adhesion and DNA/chromatin-related processes, including three histone methyltransferases (SET7,9 and 10), histone H3, and heterochromatin protein 1. Treatment with **28b** also reduced the expression levels of several Api-AP2 transcription factor family members, which are crucial in regulating the transition from ABSs to gametocytes, including Api-AP2-O3, a transcription repressor that regulates ookinete formation. Among the transcripts with increased abundance, the authors found another JmjC family member, *JmjC2*, and gametocyte-associated proteins, gametocyte development 1 (*gdv1*) and male gamete gene 1 (*mdv1*). Hence, the lack of removal of heterochromatic H3K9me3 in **28b**-treated parasites results in the parasite’s inability to prepare for gametogenesis because of gene silencing [30].

The 2-(arylcarboxamido)benzoic acid derivatives **29a**–**c** (Figure 6B), identified during a hit-to-lead optimization of inhibitors of human KDM4B (JMJD2B), displayed potent activity against late-stage gametocytes of the *P. falciparum* NF54 strain (IC_50_ = 9.34, 77.19, and 55.13 nM, respectively), with **28b** displaying an IC_50_ value of 74.18 nM in the same assay [73]. Since the only difference between **29a** and **29b** is the position of the fluorine atom on the benzoic acid moiety, the data suggest that the presence of fluorine at C5 of benzoic acid may be essential for compound activity. Differently from **29a** and **29b**, **29c** has a methyl group at C3 of the benzoic acid moiety and a *para*-tolyl group at C2 of the benzamide portion. These two substitutions seem to partially compensate for the absence of the fluorine atom. Docking studies suggested that **29a** may potentially inhibit *Pf*Jmj3. However, only a marginal increase in H3K9me3 levels was observed in late-stage gametocytes treated with **29a**, in contrast to previous results obtained with **28b** [30]. The replacement of the amide core with a sulfonamide led to an overall loss of activity. Compounds **29d** and **29e** (bearing a fluorine atom in the same position as **29a**) were the most potent compounds of the series, with IC_50_ values for their activity against late-stage gametocytes of 533 and 439 nM, respectively [73]. None of the tested compounds showed any appreciable activity on ABS parasites, with IC_50_ values > 10 µM. These compounds showed 38–55% inhibition of male gamete exflagellation at 2 µM, with **28b** being more potent in this instance (93% inhibition). Moreover, **29a**, **29b**, and **29c** were assessed in human HepG2 cells, showing <7% growth inhibition at 50 µM, and exhibited the highest human microsomal stability, with half-lives longer than 150 min. Finally, the most active compound, **29a**, showed solubility issues, while its analog **29c**, although 6-fold less potent, was >17-fold more soluble [73].

### 3.3. Antiplasmodial Transmission-Blocking Compounds Inhibiting Plasmodium Kinases

**Figure 7:**
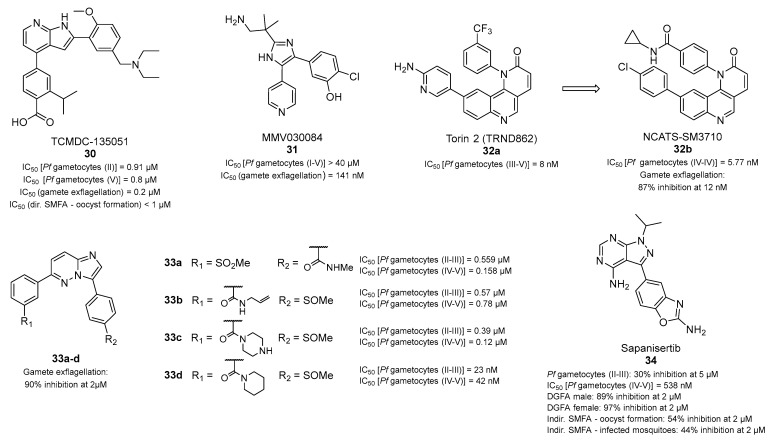
Structures and transmission-blocking activities of kinase inhibitors **30**, **31**, **32a**,**b**, **33a**–**d**, and **34**.

Recently, *P. falciparum* cyclin-dependent-like kinase (*Pf*CLK3), a protein kinase involved in pre-mRNA processing, has been indicated as a valid target for malaria treatment, as it is essential for not only ABS parasite but also sexual-stage parasite development [74]. Specifically, Mahindra et al. identified TCMDC-135051 (**30**, Figure 7) as a nanomolar inhibitor of *Pf*CLK3 (IC_50_ values of 4.8 nM [74] or 40 nM, depending on the study [75]; the discrepancy may have been due slight differences in the assay setup), with selectivity over its paralog *Pf*CLK1, its human ortholog PRPF4B, and the closely related human kinase CLK2. Compound **30** impaired *P. falciparum* 3D7 viability at the ring to trophozoite and trophozoite to schizont stages, showed submicromolar parasiticidal activity in asexual-stage *P. falciparum Pf*2004 strain, and inhibited the liver invasion of *P. berghei* in mice. Notably, **30** also impaired the viability of stage II *Pf*2004 gametocytes (IC_50_ = 0.91 μM) and exerted a concentration-dependent decrease in stage V gametocytes (IC_50_ = 0.8 μM). Additionally, **30** caused a decrease in exflagellation (IC_50_ = 0.2 μM) and reduced the prevalence of oocysts in the gut of infected mosquitoes with an IC_50_ value of 0.8 μM in an indirect SMFA [74].

The trisubstituted imidazole MMV030084 (**31**, Figure 7) has been recently indicated to possess multistage and transmission-blocking antimalarial potential. Compound **31** potently impaired *P. falciparum* liver-stage invasion (IC_50_ = 199 nM) and showed low cytotoxicity against human HepG2 cells. Compound **31** also targeted ABS development (IC_50_ = 120 nM), where it primarily affected schizonts and inhibited parasite egress from infected RBCs [76]. Compound **31** also inhibited male gametogenesis in vitro but did not kill mature gametocytes directly. In line with this, **31** was ineffective against both early- and late-stage *P. falciparum* NF54 gametocytes (IC_50_ > 40 μM), while it inhibited male gamete exflagellation (IC_50_ = 141 nM) when gametocytes were stimulated to develop into male gametes in the presence of the compound. This activity was lost when **31** was washed out before gamete stimulation, consistent with the lack of gametocytocidal activity. Comprehensive target identification and validation studies, including chemoproteomics, conditional knockdown, and molecular modeling experiments, highlighted the plasmodial cGMP-dependent protein kinase (*Pf*PKG) as the primary target of **31**. *Pf*PKG showed the lowest K_D_^app^ values (9–16 nM, depending on the assay) and the largest growth defect and sensitization to **31** in conditional knockdown experiments. In addition, **31** docked well into the ATP-binding pocket of *Pf*PKG and inhibited recombinant *Pf*PKG activity with an IC_50_ value of 0.4 nM.

The benzo[*h*][1,6]naphthyridin-2(1*H*)-one derivative torin 2 (**32a**, Figure 7) is a known inhibitor of the mechanistic target of rapamycin (mTOR) endowed with a subnanomolar efficacy against *P. falciparum* life-cycle stages (IC_50_ of 8 nM against stage III–V gametocytes) and also acted as a potent in vivo transmission-blocking agent in a mouse model of *P. berghei* infection [77,78]. Based on its structure, Krishnan et al. developed a series of analogs that led to NCATS-SM3710 (**32b**, Figure 7), characterized by improved safety, solubility, and metabolic stability [79]. Compound **32b** displayed an IC_50_ of 0.38 nM against ABS *P. falciparum* NF54 and potent gametocytocidal activity (IC_50_ = 5.77 nM in stage IV–V gametocytes) and could block mature gametocyte production (stage III–IV gametocytes), resulting in complete clearance after 96 h at 2 nM. At 12 nM, **32b** reduced exflagellation by up to 87% after 20 h of incubation. Furthermore, the evaluation of mosquitoes fed with **32b**-treated *P. berghei* showed complete inhibition of oocyst development in the mosquito midgut. In addition, in vitro ookinete assays using blood from *P. berghei-*infected mice highlighted that treatment with **32b** (2 × 40 mg/kg doses at 2.5 h interval) blocked ookinete development 24 h post-blood meal, confirming its transmission-blocking efficacy. Multiple lines of evidence obtained via genetic studies on **32a-** and **32b**-resistant parasites suggest that *Pf*PI4Kβ is a target for both compounds. In line with this, **32b** inhibited recombinant *Pf*PI4Kβ with an IC_50_ value of 2.0 nM, which is consistent with its in vitro activity against parasites.

Cheuka et al. recently developed 3,6-diphenylated imidazopyridazines (Figure 7) showing promising multistage antiplasmodial activity. Among these derivatives, four compounds displayed submicromolar inhibition of both the asexual and sexual stages of *P. falciparum* NF54 development [80]. These include **33a**, presenting a *N*-methylcarboxamide group on C3 and a 3-methylsulfinylphenyl moiety at C6, **33b**, **33c**, and **33d**, bearing a 4-methylsulfinylphenyl moiety at C3 and different *N*-(cyclo)alkylcarboxamides at C6 (Figure 7) [80]. Specifically, **33a** displayed IC_50_ values of 0.559 and 0.158 µM against stage II–III and IV–V gametocytes, respectively. Compounds **33b** [IC_50_ (II–III) = 0.57 μM; IC_50_ (IV–V) = 0.78 μM] and **33c** [IC_50_ (II–III) = 0.39 μM; IC_50_ (IV–V) = 0.12 μM] were also active at submicromolar concentrations against both stages, while **33d** possessed IC_50_ values in the nanomolar range [IC_50_ (II–III) = 23 nM; IC_50_ (IV–V) = 42 nM]. Additionally, these compounds prevented more than 90% of male gamete exflagellation at 2 μM. Finally, all compounds displayed >90% inhibition against *P. vivax* PI4K at 0.1 µM, with **33c** also inhibiting *Pf*PKG (95% at 10 µM). However, **33a**, **33c**, and **33d** also displayed > 90% inhibition of human PI4Kβ at 1 µM (**33b** was not tested), which highlights a possible risk of off-target cytotoxicity.

Sapanisertib (**34**, Figure 7), a pyrazolopyrimidine acting as an ATP-competitive inhibitor of the mTOR complexes 1 and 2, was recently indicated to possess multistage antiplasmodial activity. Specifically, **34** displayed prophylactic liver-stage activity (IC_50_ = 134 nM) coupled with low HepG2 cytotoxicity, ABS activity both in vitro (IC_50_= 76 nM) and in vivo, and transmission-blocking activity [81]. Compound **34** showed great potency against late-stage (IV–V) gametocytes (IC_50_ = 538 nM) rather than early-stage (II–III) gametocytes (30% inhibition at 5 μM). Additionally, **34** consistently inhibited male and female gamete formation (89% and 97% inhibition at 2 μM, respectively) in an in vitro exflagellation inhibition assay (EIA) and a female gamete activation assay (FGAA). Furthermore, in an indirect SMFA, the incubation of **34** (at 2 μM) with stage V gametocytes for 48 h before mosquito feeding reduced both oocyst formation and the prevalence of infected mosquitoes by 54% and 44%, respectively, relative to controls. Chemoproteomics and biochemical assays identified *Pf*PI4Kβ (IC_50_ = 4 nM) and *Pf*PKG (IC_50_ = 20 nM) as the most likely targets of **34**. Finally, knockdown and in vitro resistance selection studies suggested that the primary target of sapanisertib is *Pf*PI4Kβ in asexual blood-stage parasites.

### 3.4. Aminoacyl-tRNA Synthetase Inhibitors

Protein synthesis is a critical step in the life cycle of all cells. As in other cellular organisms, *Plasmodium* spp. rely on the activity of aminoacyl-tRNA synthetases (aaRSs), which ensure the formation of aminoacyl-tRNAs in the translation process. aaRSs catalyze the formation of the final “charged” aminoacyl-tRNA in a two-step reaction passing through an aminoacyl-adenylate intermediate [82]. *P. falciparum* has a total of 36 aaRSs located in different cellular compartments, such as the cytoplasm, mitochondria, and apicoplast [82]. *Plasmodium* aaRSs possess additional domains that differentiate them from their human counterparts and are also involved in non-canonical functions, such as the host immune response and DNA-damage response [83,84]. Phenylalanyl-tRNA synthetase (PheRS) was proven to be a key factor for the survival of parasites in multiple steps, including transmission stages. Indeed, *Plasmodium* PheRS is the only enzyme that is imported into parasite mitochondria, whose activity increases during gametocytogenesis and becomes essential in mosquito stages [84,85].

**Figure 8:**
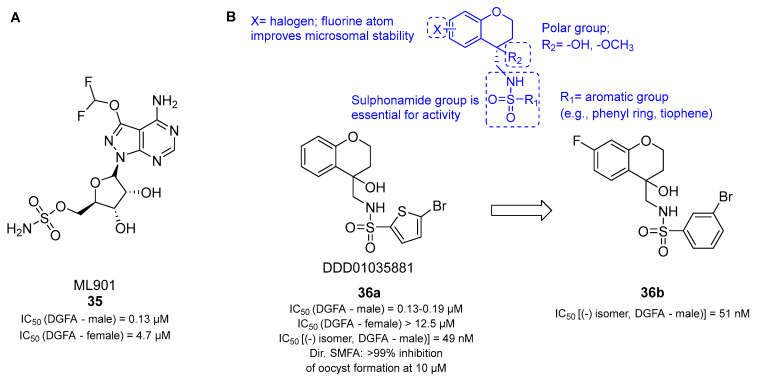
(**A**) The structure and transmission-blocking activity of the YRS inhibitor ML901 (**35**). (**B**) Structures, transmission-blocking activities, and SARs of the most relevant N-4HCS **36a**,**b**.

Recently, Xie et al. reported the adenosine-5′-sulfamate (AMS) derivative ML901 (**35**, Figure 8A), which inhibits the ABS with nanomolar activity and impairs gamete development (Figure 8A) [86]. Indeed, a DGFA showed that **35** could inhibit both male and female gamete formation, with IC_50_ values of 0.13 and 4.7 μM, respectively (Figure 8A). Compound **35** acts by interrupting protein synthesis through the covalent inhibition of *P. falciparum* tyrosine-tRNA synthetases (*Pf*YRSs). Interestingly, the authors demonstrated that the conjugate **35**-Tyr can inhibit both parasite and human YRSs (hYRS). Nevertheless, the first reaction step that leads to the formation of the **35**-Tyr conjugate can occur only in *Pf*YRS. Therefore, it can be assumed that **35** shows pro-drug-like behavior [86,87]. Compound **35** is substituted at position C3 by a difluoromethoxy group, and it was the only AMS derivative that displayed selective activity for *P. falciparum* over mammalian cells (selectivity index between 800 and 5000). Due to its chemopreventive and submicromolar transmission-blocking effects, favorable PK, and single-dose efficacy in vivo, 35 is a good candidate for a hit-to-lead campaign. Structural modifications are still required to minimize off-target interactions with the human E1 enzyme ATG7, a common target of all AMS analogs. Xie et al.’s findings not only brought up new possibilities for the design of the next generation of antimalarial drugs but also uncovered *Pf*YRS as a new drug target for the development of drugs targeting transmission stages. Overall, it is conceivable that future antimalarial drug discovery will also focus on aaRS inhibition since these enzymes have been shown to play more than one important role at different levels in the parasite life cycle [82,88,89]. To the best of our knowledge, only a small number of the 36 aaRSs of *Plasmodium* spp. have been studied, and only two chemical classes, byciclic azetedines [90] and AMS analogs [86], targeting *Pf*PheRS and *Pf*YRS, respectively, have been identified as potential transmission-blocking agents. This strongly suggests that advancements in the research of *Plasmodium* aaRSs may result in novel chemotypes that could offer alternative ways to prevent malaria transmission.

### 3.5. Pfs16 Inhibitors

Pfs16 is a key marker of early *P. falciparum* gametocytes and is highly expressed until the gamete stage, showing similar levels between male and female parasites. Although the exact function of this protein is still unknown, it is found in the parasitophorous vacuole membrane (PVM) and is consistently connected with the ability to develop mature gametocytes in vitro and with male gametocyte exflagellation [91,92,93]. Pfs16 is not required for parasite survival but plays an important role in sexual differentiation [94], thereby being a viable drug target.

A recent screening of 70,000 compounds from the Global Health Chemical Diversity Library from the University of Dundee showed that *N*-((4-hydroxychroman-4-yl) methyl) sulphonamide (N-4HCS) derivatives block *Plasmodium* male gamete formation through Pfs16 inhibition and can target early gamete formation before DNA replication and exflagellation with nanomolar potency [39,95]. Among the tested molecules, DDD01035881 (**36a**, Figure 8B) was the most potent one. DGFA experiments yielded IC_50_ values against male and female gamete formation of 0.19 μM and >12.5 μM, respectively. Transmission-blocking activity was confirmed in a direct SMFA (>99% inhibition of oocyst formation at 10 μM). Moreover, **36a** reduced oocyst formation in *P. berghei-*infected mice by 99.6% when administered at 50 mg/kg 30 min before mosquito feeding. Using photoaffinity labeling and pulldown to identify the target and a cellular thermal shift assay (CETSA) and cellular analysis of treated parasites to validate the target, Yahiya et al. [95] identified Pfs16 as a putative target for **36a**. When **36a** was used in a specific time window (0–5 min after microgametogenesis induction), it could block parasite egress. This was associated with the specific binding of the compound to the capping sequence of Pfs16, which was suggested to play a crucial role during cytoskeletal rearrangements and PVM degradation and the consequent parasite egress from the erythrocyte and microgamete formation. In line with this, immunofluorescence (IF) microscopy or electron microscopy (EM) showed that the incubation of **36a** (5 μM) with gametocytes could disrupt cytoskeletal, nuclear, and parasitophorous food vacuole structures. Their analysis not only confirmed *Pf*s16 as a valuable transmission-blocking target but also highlighted its involvement in the microgametogenesis process. Although **36a** is non-toxic (<50% HepG2 inhibition at 10 μM), soluble in water (>250 μM), and not subject to mouse liver microsome metabolism, its poor half-life (~90 min) restrains its advancement to clinical investigations. In an effort to obtain N-4HCSs with longer half-lives, Rueda-Zubiaurre et al. performed a SAR study, which led to derivative **36b** (Figure 8B) [96]. SAR analysis showed that the sulphonamide R_1_ group is essential for activity and cannot be modified or replaced with an amide group. The R_2_ position allows the presence of an aromatic ring, such as a tiophene or phenyl ring, that could be appropriately substituted with halogens to enhance metabolic stability, but not with bigger electron-withdrawing groups. Furthermore, the chromane ring is not amenable to size changes and allows few changes with fluorinated derivatives to improve the half-life of these analogs. A DGFA indicated that in the cases of both **36a** and **36b**, the active enantiomer is the (-) isomer, with IC_50_ values of 46 and 51 nM, respectively.

### 3.6. Acetyl Coenzyme a Synthesis Inhibitors

Acetyl coenzyme A (Ac-CoA) is one of the primary sources that eukaryotic cells (i.e., *Plasmodium* spp.) use to provide the acetyl moieties needed for cell growth regulation and proliferation [97]. In *P. falciparum*, Ac-CoA levels are mostly sustained by the activity of two enzymes, the mitochondrially localized branched-chain keto-dehydrogenase (BCKDH) complex and cytosolic/nuclear Ac-CoA synthetase (ACS), which convert glucose-derived pyruvate and acetate into Ac-CoA, respectively [98,99,100,101,102]. ACS was found to be indispensable for parasite survival in blood stages. Indeed, its inhibition or knockdown led to a decrease in acetyl-CoA levels and impairment in the epigenetic regulation of gene expression, which has always resulted in a cell growth defect [101,102,103]. Furthermore, acetyl-CoA is required by *P. falciparum* during the asexual cycle and gametocyte development [101,104]. Moreover, pantothenate (vitamin B5) is essential for *Plasmodium* viability since it is required for CoA biosynthesis.

**Figure 9:**
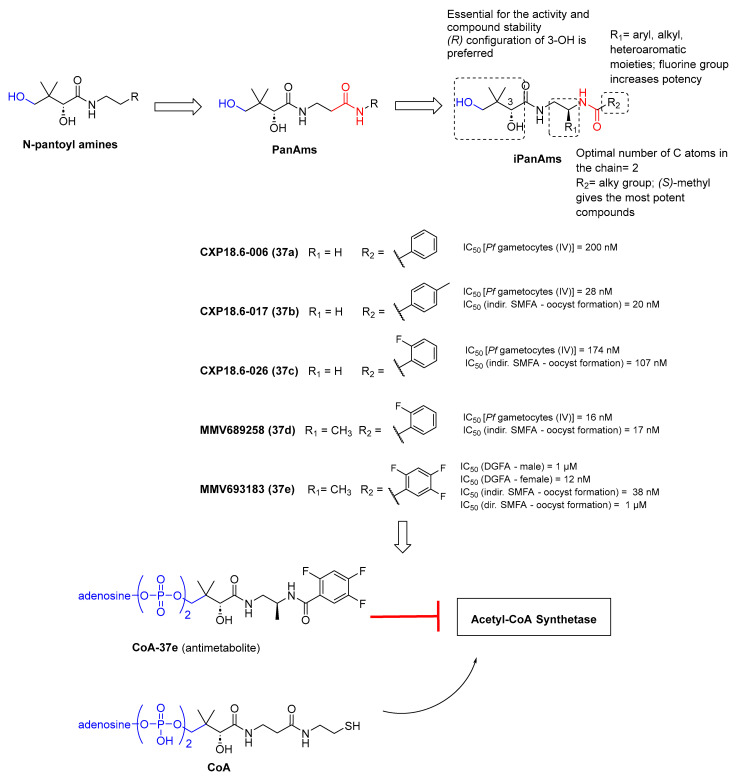
Development, chemical structures, transmission-blocking activities, and mode of action of iPanAms **37a**–**e**.

Schalkwijk et al. developed a series of pantothenamide analogs characterized by an inverted amide bond (iPanAms) (Figure 9) [105,106]. Among them, compounds CXP18.6-006 (**37a**), CXP18.6-017 (**37b**), CXP18.6-026 (**37c**), and MMV689258 (**37d**) exhibited antiplasmodial activity against both the ABS and gametocytes of the *P. falciparum* NF54 strain in the low- to mid-nanomolar range [106]. Specifically, the four compounds displayed IC_50_ values of 200, 28, 174, and 16 nM, respectively, against stage IV gametocytes. In an indirect SMFA, the incubation of increasing concentrations of **37b**–**d** (Figure 9) with stage V gametocytes from the luminescent reporter strain NF54-HGL for 24 h before mosquito feeding blocked oocyst formation with IC_50_ values of 20, 107, and 17 nM, respectively. Although the proposed mechanism involved the inhibition of *P. falciparum* pantothenate kinase 1 (*Pf*PANK1), the compounds only inhibited the protein in the sub-/low-micromolar range, suggesting that *Pf*PANK1 is not their primary target. Further experiments confirmed that all compounds are converted into CoA antimetabolites, which may interfere with CoA-dependent pathways. Compound **37d** was also tested in mouse models, where it displayed good PK properties and could decrease parasitemia 3 days after infection by 77% to 99.9% with a single dose of 25 to 200 mg/kg. Notably, in vivo studies showed that after a brief exposure to **37d**, parasitemia continued to decrease for several days before growth resumed. The continued elimination of parasites after the point where **37d** plasma concentrations were undetectable may be attributed to the “post-antibiotic effect”, which is likely associated with the accumulation of CoA antimetabolites. The most advanced iPanAm is MMV693183 (**37e**, Figure 9), which has nanomolar anti-ABS activity against both *P. falciparum* and *P. vivax*, as well as nanomolar potency against *P. falciparum* gametocytes, with higher potency against female gametocytes (IC_50_ values of 1 μM and 12 nM against male and female gamete formation, respectively) [105]. In an indirect SMFA, **37e** inhibited the transmission of parasites in *Anopheles stephensi* mosquitoes with an IC_50_ value of 38 nM, while it did not kill mosquito-stage parasites in a direct SMFA at a concentration of 1 μM. These data indicate that **37e** can only prevent the transmission of parasites to mosquitoes by targeting gametocytes [105].

### 3.7. Transmission-Blocking Compounds Altering Microtubule Assembly, Plasmepsins IX and X, and Pf20S Proteasome

**Figure 10:**
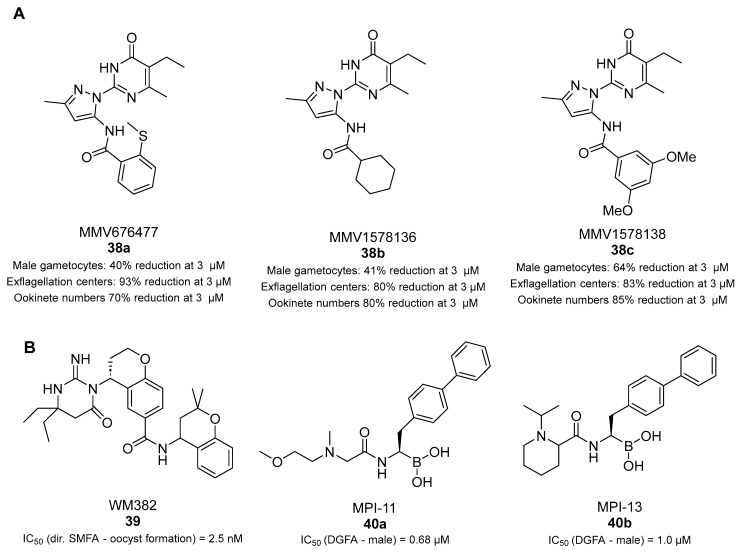
(**A**) Structures and transmission-blocking activities of compounds **38a**–**c** targeting microtubule assembly. (**B**) Structures and transmission-blocking activities of compounds inhibiting plasmepsins IX and X (**39**) and *Pf*20S proteasome (**40a**,**b**).

*P. falciparum* tubulin (*Pf*Tubulin) proteins represent promising drug targets for antiplasmodial therapy because plasmodial microtubules play a crucial role during parasite proliferation, growth, and transmission. Kumari et al. evaluated the antimalarial activity of compounds belonging to the MMV Pathogen Box for their capability of altering microtubule dynamics [107]. Surface plasmon resonance (SPR) assays showed that the 2-pyrazolylpyrimidinone MMV676477 (**38a**, Figure 10A) and its derivatives interact with either *Pf*α- or *Pf*β-tubulin, or both, with **38a** possessing a *K_D_* value of 27.7 μM for *Pf*α-tubulin while not interacting with *Pf*β-tubulin, as confirmed by a CETSA. Moreover, these compounds were shown to disturb *Pf* microtubule assembly in vitro by disrupting *Pf*Tubulin polymerization and altering *Pf*β-tubulin GTPase activity. Among the tested molecules, **38a**, MMV1578136 (**38b**), and MMV1578138 (**38c**) (Figure 10A) inhibited the growth of ABS *P. falciparum* 3D7, RKL-9 (chloroquine-resistant), and R539T (artemisinin-resistant) with IC_50_ values in the submicromolar range and displayed low cytotoxicity against human HepG2 cells. Furthermore, cultures of blood from *P. berghei*-infected mice incubated with **38a**–**c** for 1 h at 3 μM displayed reductions in mature gametocytes of 40%, 41%, and 64%, respectively. Since microtubule dynamics is crucial for the exflagellation of male gametocytes, the results of ex vivo exflagellation assays with *P. berghei*-infected RBCs obtained from infected mice treated with the three compounds at 3 μM showed significant reductions in the number of exflagellation centers of approximately 93%, 80%, and 83%, respectively. Moreover, treatment of *P. berghei*-infected RBCs with **38a**–**c** led to decreases in ookinete numbers of 70%, 80%, and 85%, respectively, along with incomplete development and morphological maturation of ookinetes.

Plasmepsins IX and X (PMIX and PMX) are aspartic proteases that have recently been identified as potential new targets. Inhibitors of these proteases block parasite egress and invasion [108,109]. Favuzza et al. identified WM382 (**39**, Figure 10B) through the screening and optimization of an aspartic protease inhibitor library. Compound **39** was shown to disrupt multiple stages of the *P. falciparum* life cycle [110] and to target plasmodial ABSs both in vitro and in vivo. It inhibited PMX with an IC_50_ value of 0.06 nM, and knockdown and CETSA experiments indicated both PMIX and PMX as its possible targets. Notably, a direct SMFA showed that treatment of *P. falciparum* gametocytes with increasing concentrations of **39** before mosquito feeding could block oocyst formation at a concentration of 2.5 nM.

Xie et al. identified a series of amino-amide boronates that are potent and specific inhibitors of the *P. falciparum* 20S proteasome (*Pf*20S) β5 active site and exhibit fast-acting antimalarial activity. Among them, two amino-amide boronates showed promising transmission-blocking activity. The biphenyl-containing MPI-11 (**40a**) and MPI-13 (**40b**) (Figure 10B) exhibited IC_50_ values against *Pf*20S β5 of 5 and 12 nM, respectively [111]. Both compounds were more than 14-fold selective over the human 20S β5c subunit while being only 2-fold selective over the 20S β5i present in immune cells. Both compounds were tested through a DGFA and inhibited male exflagellation with IC_50_ values of 0.68 μM and 1.0 μM, respectively, and exhibited nanomolar inhibition of *P. falciparum* 3D7 asexual-stage parasites. Compound **40a** also inhibited the exoerythrocytic stage of *P. berghei* in a human hepatoma cell line, prevented hepatic merozoite formation, and blocked the development of *P. falciparum* NF54 schizonts in primary human hepatocytes, and hepatic toxicity was not apparent. Additionally, both **40a** and **40b** exhibited limited toxicity against HepG2 cells [111].

### 3.8. Drug Repurposing as an Approach to Developing Transmission-Blocking Compounds

**Figure 11:**
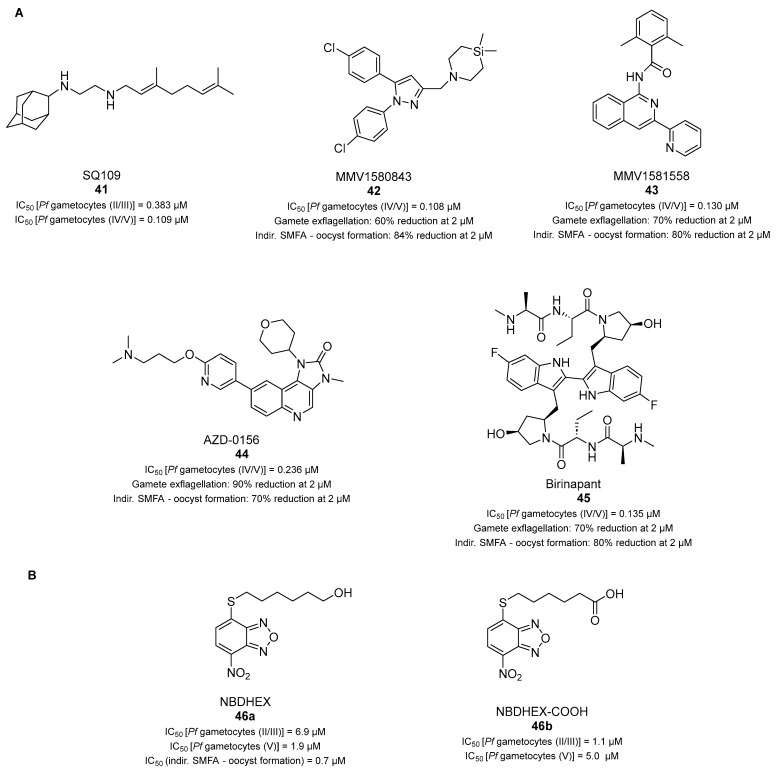
Structures and transmission-blocking activities of compounds **41**–**45** (**A**) and NBDHEX (**46a**) and its metabolite NBDHEX-COOH (**46b**) (**B**).

Recent studies [30] have demonstrated that the antitubercular clinical candidate MmpL3 inhibitor SQ109 (**41**, Figure 11A) inhibits parasite viability in stage IV–V gametocytes (IC_50_ = 0.109 µM). This compound is also active against stage II–III gametocytes and asexual parasites, although to a lesser extent (IC_50_ values of 0.383 and 1.39 µM, respectively) [30]. The gametocytocidal activity of **41** was similar after 12, 24, or 48 h of exposure, indicating that the effect was present within 12 h of incubation with gametocytes. Furthermore, **41** generated two resistant mutants bearing mutations in the *P. falciparum* V-type H^+^-ATPase (*Pf*vapA), a druggable protein in *Plasmodium* targeted by another antimalarial candidate class, triaminopyrimidine (TAP).

In the same study, the rimonabant derivative MMV1580843 (**42**, Figure 11A), also reported as an MmpL3 inhibitor [112], was shown to impair the viability of stage IV–V *P. falciparum* NF54 gametocytes with an IC_50_ value of 0.108 µM, while its potency against ABS parasites was lower (IC_50_ = 0.78 µM) [112]. Moreover, **42** reduced male gamete exflagellation by 60% at 2 µM and decreased oocyst formation by 84% in an indirect SMFA performed by feeding *A. coluzzi* mosquitoes with compound-treated gametocyte cultures (48 h treatment at 2 μM). A similar profile was observed for the isoquinoline-based A3 adenosine receptor inhibitor MMV1581558 (**43**, Figure 11A), exhibiting an IC_50_ value of 0.130 µM against stage IV–V gametocytes, a ~70% reduction in male gamete exflagellation at 2 µM, and an ~80% decrease in oocyst formation in an SMFA performed as described above [30]. Moreover, the Ataxia Telangiectasia Mutated kinase inhibitor AZD-0156 (**44**, Figure 11A) [113] and the peptidomimetic antagonist of the inhibitor of apoptosis (IAP) proteins birinapant (**45**, Figure 11A) [114] exhibited multistage activity by targeting the ABS and liver-stage (IC_50_ values in the low- to sub- micromolar range) as well as stage IV–V gametocytes [IC_50_(**44**) = 0.236 µM; IC_50_(**45**) = 0.135 µM]. Compounds **44** and **45** (both at 2 µM) also decreased male gamete exflagellation by ~90% and ~70%, respectively, and reduced oocyst formation in an indirect SMFA by ~70% and ~80%, respectively [30].

The nitrobenzoxadiazole derivative 6-((7-nitrobenzo[c][1,2,5]oxadiazol-4-yl)thio)hexan-1-ol (NBDHEX, **46a**) (Figure 11B) is a known inhibitor of the human glutathione *S*-transferases (GSTs) P1-1 and M2-2 (GSTP1-1 and GSTM2-2, respectively) and was previously identified as a potent cytotoxic agent against murine and human cancer cells and against the protozoan parasite *Giardia duodenalis* [115,116,117]. Recently, **46a** and its carboxylic acid metabolite (NBDHEX-COOH, **46b**) (Figure 11B) have been reported as antiplasmodial transmission-blocking agents [118]. Both **46a** and **46b** impaired the viability of stage II–III [IC_50_(**46a**) = 6.9 µM, IC_50_(**46b**) = 1.1 μM] and stage V [IC_50_(**46a**) = 1.9 µM, IC_50_(**46b**) = 5.0 µM] gametocytes in a luciferase assay in the *P. falciparum* strain NF54 pfs16-GFP-PyLUC while being slightly less active against asexual parasites [IC_50_(**46a**) = 7.9 µM, IC_50_(**46b**) = 16.4 μM]. The transmission-blocking efficacy of **46a** was confirmed by an indirect SMFA, showing that the inhibitor impaired oocyst formation in mosquitoes with an IC_50_ value of 0.7 μM. Notably, **46a** retained significant cytotoxic activity in human VERO cells after 48 h of exposure (IC_50_ = 7.97 μM), while **46b** was less cytotoxic (IC_50_ > 10 μM). Mechanistically, **46a** was shown to be ~100-fold less potent against *Pf*GST than GSTP1-1, indicating that it is not its main target. Instead, mass spectrometric analysis revealed the formation of covalent adducts between the 7-nitro moiety of **46a** and cysteine residues present in five gametocyte proteins [glyceraldehyde-3-phosphate dehydrogenase (GAPDH), 14-3-3 isoform I (14-3-3I), cell division cycle protein 48 (Cdc48), α-tubulin 2, and 60S ribosomal protein L7a (eL8)]. Hence, **46a**, and likely **46b**, may alter the activity of these enzymes, thereby disrupting pathways that are vital for the parasite.

### 3.9. Multistage Active Drugs with Unknown Targets

**Figure 12:**
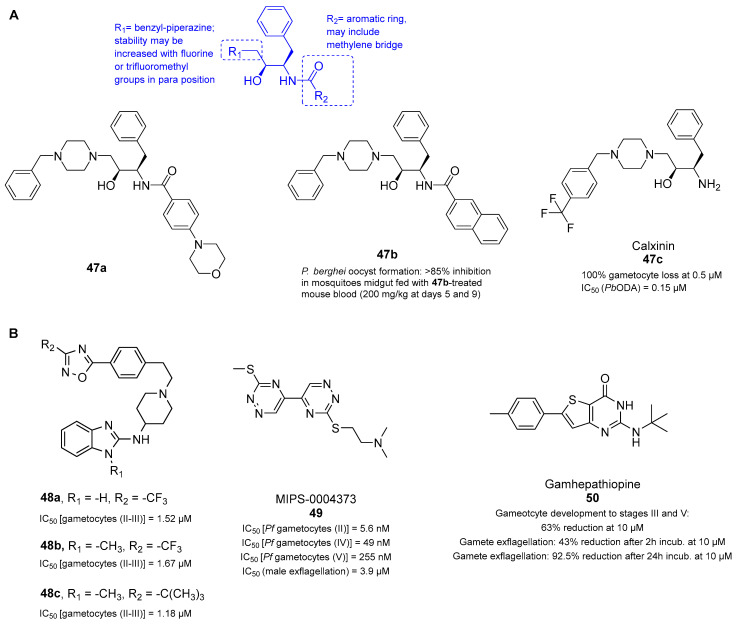
(**A**) Structures, SARs, and transmission-blocking activities of compounds **47a**–**c**. (**B**) Structures and transmission-blocking activities of compounds **48a**–**c**, **49**, and **50**.

Hydroxyethylamine (HEA) has been identified by Brijesh Rathi et al. as a promising pharmacophore for the development of many antimalarial drugs [119,120,121,122]. Some symmetrical and peptidomimetic HEAs have been documented as potent inhibitors of PMs [123], which represent an interesting target for malaria transmission-blocking strategies, as previously mentioned. After the exploration of 13 HEA-piperazine analogs, compounds **47a** and **47b** (Figure 12) exhibited the lowest IC_50_ values against the *P. falciparum* ABS (0.21 μM and 0.15 μM, respectively), with **47b** showing a better safety profile both in vitro and in vivo. Both compounds were active against chloroquine- and artemisinin-resistant strains and were strongly efficient in clearing *P. berghei* parasites in infected mice at a dosage of 50 mg/kg and at 30 mg/kg in combination therapy with artesunate (**47b**:artesunate in a 5:3 ratio). Compound **47b** showed transmission-blocking potential in a *Pb*ODA at sub-micromolar concentrations (0.05, 0.15, and 0.45 μM). Its activity was further validated by feeding *Anopheles stephensi* mosquitoes with *P. berghei*-infected mice blood following treatment with **5d** (200 mg/kg at days 5 and 9) or DMSO. Their results showed a >85% reduction in the number of oocysts formed in the mosquito midgut. Overall, the introduction of piperazine to the HEA backbone is favorable for activity and drug-like properties. Compounds **47a** and **47b** proved that the introduction of heterocyclic rings (e.g., morpholine) or aromatic rings (e.g., naphthalene) can be beneficial for compound activity and drug-like properties (e.g., longer half-life). In silico docking studies predicted that **47a**,**b** have high binding affinities with PM II, thereby providing a putative target for this class of compounds. However, further work is warranted to validate this hypothesis and better understand the drug binding [124].

A more recent study identified the HEA-based piperazine compound calxinin (**47c**, Figure 12A) as a promising lead antimalarial compound with potent multistage activity [125]. Unlike the HEA analogs previously described, it contains a primary amine. Compound **47c** was found to target the asexual stages of *P. falciparum* with nanomolar potency (IC_50_ values of 88–135 nM) and proved to be even more active against liver stages (79 nM in vitro and 30% inhibition at 10 mg/kg in vivo). Compound **47c** was also active against early-stage gametocytes, causing 100% mature gametocyte loss at 0.5 μM. Encouraging transmission-blocking activities were similarly obtained in the *Pb*ODA. Indeed, **47c** inhibited ookinete development with an IC_50_ value of 0.15 μM. However, likely due to poor PK, it was not fully effective at a single dose of 50 mg/kg when given to cure the mouse infection, only reducing 27.4% of parasitemia. Compound **47c** was shown to interfere with *Plasmodium* Ca^2+^ homeostasis, causing cell swelling/deformation in sexual and mosquito stages. However, resistant mutant selection experiments failed to provide mutant parasites or determine the specific target of this compound. Compound **47c** is a promising antimalarial drug candidate due to its ability to target many parasite life stages, including the liver, asexual, sexual, and sporogonic stages [125]. Collectively, these results indicate that HEAs can be effective multistage antimalarials with the potential to block the transmission of *P. falciparum* but still need PK optimization.

Mambwe et al. recently reported a series of astemizole analogs, among which the 1,2,4-oxadiazole derivatives **48a**–**c** (Figure 12B) presented mid-nanomolar activity in the asexual stages of *P. falciparum* while exhibiting low-micromolar gametocytocidal activity in stage I–III gametocytes [126]. Compared to astemizole, the compounds seem to have a lower cardiotoxicity risk because they showed a lower affinity for the human *Ether-à-go-go*-Related Gene (hERG) K^+^ channel [IC_50_ (hERG) = 0.63–1.35 μM, >1000-fold higher selectivity compared to astemizole]. Specifically, the compounds exhibited IC_50_ values against stage I–III gametocytes of 1.52, 1.67, and 1.18 µM, respectively, while they did not show significant activity against stage IV–V gametocytes at 1 or 5 µM. Furthermore, **48a** and **48b** showed 99.5% and 90% reductions in parasitemia in mice (following a regimen of 50 mg/kg/day for 4 days), along with good PK properties and metabolic stability.

Ellis et al. recently reported the bis-1,2,4-triazine MIPS-0004373 (**49**, Figure 12B) as a potent multistage and antiplasmodial compound, although its mode of action is still unknown [127]. Beyond showing nanomolar activity against all asexual *P. falciparum* blood stages (IC_50_ values < 100 nM), the extended in vitro exposure to compound **49** indicated a low propensity for the emergence of resistance. Moreover, **49** exhibited gametocytocidal activity against stage II gametocytes with an IC_50_ value of 5.6 nM. However, its activity decreased as gametocytogenesis advanced, with higher IC_50_ values measured against stage IV and stage V gametocytes (49 and 255 nM, respectively). Additionally, action against mature gametocytes was restricted to suppressing male gametogenesis since **49** showed extremely weak activity against female gametocytes (IC_50_ > 25 μM) and low-micromolar activity in the male exflagellation assay (IC_50_ = 3.9 μM). Finally, **49** efficiently cleared an established *P. berghei* infection in vivo (at a dose of 64 mg/kg/day), with efficacy and recrudescence profiles similar to those of chloroquine used at the same dose.

The thienopyrimidine gamhepathiopine (**50**, Figure 12B) exhibited multistage activity, with mid-nanomolar potency against the blood stages of *P. falciparum* [128] and submicromolar activity against the liver stages of *P. falciparum*, *P. yoelii*, and *P. cynomolgi*, accompanied by gametocytocidal activity [129]. At 10 µM, **50** diminished the conversion rate of asexual parasites into gametocytes by 87.8% and reduced gametocyte development to stage III or stage V by ~63% in both cases. Moreover, **50** blocked male exflagellation with percentages of exflagellation of 57% and 7.5% after 2 and 24 h of incubation, respectively. According to these findings, **50** may prevent exflagellation by either directly inhibiting gamete development or “sterilizing” the mature gametocyte, rendering it metabolically viable but unable to produce gametes. Finally, in an in vivo mosquito feeding assay, treatment of *P. yoelii*-infected mice with **50** (50 mg/kg) hours before mosquito feeding reduced the infection rate from 82.8% to 37.1% and the mean number of oocysts from 106 to 1. Notably, treatment of *P. yoelii*-infected mice with **50** for 3 days at 50 mg/kg reduced parasitemia by 50% but had a lower influence on the infection rate (71.4%) following mosquito feeding and the mean number of oocysts per mosquito [129].

**Figure 13:**
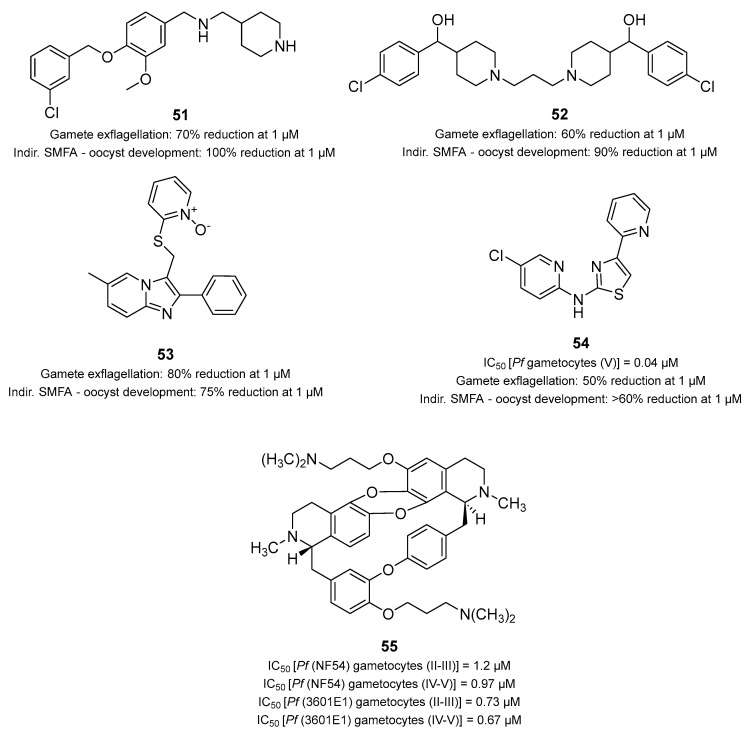
Structures and biological transmission-blocking activities of compounds **51**–**55**.

Recently, Paonessa et al. performed a high-throughput screening on *P. falciparum* gametocytes that led to the identification of five new small molecules acting as transmission-blocking compounds [130]. These include compound **51** (Figure 13), previously discovered as an ABS inhibitor [131], which was shown to decrease male gamete exflagellation by ~70% at 1 µM. Compound **51** also completely suppressed oocyst formation in an indirect SMFA performed by incubating the *P. falciparum* strain NF54-hsp70-luc with 1 µM compound for 24 h. The second most active compound in the SMFA was the symmetric 4-piperidinyl benzyl derivative compound **52** (Figure 13), which decreased oocyst intensity by 90% at 1 µM and could also reduce male gamete exflagellation by ~60% at 1 µM. The disubstituted imidazo[1,2-a]pyridine compound **53** (Figure 13) was the third most active hit in the SMFA, as it reduced oocyst intensity by 75% at 1 µM while being more active in inhibiting male gamete exflagellation (~80% inhibition at 1 µM). Finally, TCMDC-125769 (**54**, Figure 13), previously shown to target the *P. falciparum* asexual stage (IC_50_ = 1 µM) and stage V gametocytes (IC_50_ = 0.04 µM) [132], was demonstrated to inhibit male gamete exflagellation by ~50% at 1 µM. In the same indirect SMFA as above, compound **54** reduced oocyst intensity by >60% at 1 µM.

Following a screening of a chemical library of hemisynthetic derivatives of the natural product trilobine, Nardella et al. identified compound **55** (Figure 13), which was active against the ABS of the *P. falciparum* NF54 strain with an IC_50_ of 123 nM and showed good selectivity over human HepG2 cells (IC_50_ = 2.4 μM) [133]. Furthermore, **55** showed transmission-blocking activity with IC_50_ values of 1.2 and 0.97 μM against early- (II–III) and late-stage (IV–V) *P. falciparum* NF54 gametocytes, respectively. Notably, **55** was slightly more active against the Cambodian strain 3601E1 resistant to artemisinin, with IC_50_ values of 0.73 and 0.67 μM in early- and late-stage gametocytes, respectively. Moreover, the addition of compound **55** (10 μM) to the blood meal of the *Anopheles* mosquito significantly decreased the oocyst number without an increase in mosquito mortality. Moreover, **55** showed dose-dependent inhibition of parasite development in hepatocytes, indicating that **55** targets proteins expressed during multiple life-cycle stages of *P. falciparum*. Finally, **55** exhibited good results in vivo, as it reduced mouse parasitemia when administered at 20 mg/kg and increased mouse survival by 5 days. Chemical pulldown assays using a bioluminescent derivative of **55** showed that the possible pathway targeted by this compound may be the polyadenylate-binding protein 1 complex (PABP1 and partners), involved in protein translation, and/or the proliferating cell nuclear antigen (PCNA1), involved in DNA replication and DNA methylation [133]. Nevertheless, additional studies are necessary to identify the specific protein target.

### 3.10. Innovative Approaches: Atovaquone-Coated Surfaces to Block Parasite Transmission

**Figure 14:**
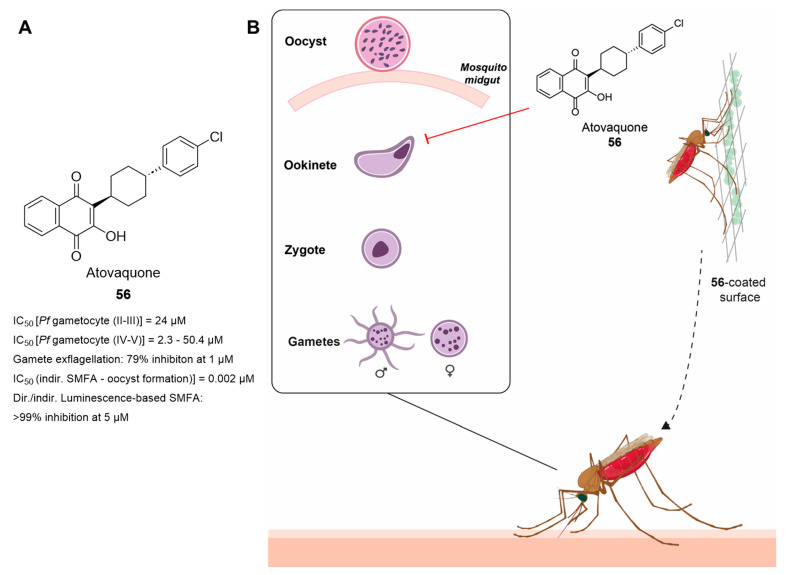
(**A**) Atovaquone (**56**) structure and transmission-blocking activities. (**B**) Schematic of the atovaquone-coated-surface approach developed by Paton et al. [145]. Created with Biorender.com.

While ABS parasites primarily produce energy through glycolysis, during early gametocytogenesis, their metabolism shifts to become more oxidative, and hence, parasites rely more on the tricarboxylic acid cycle and aerobic energy generation via the electron transport chain (ETC) [134]. This is exemplified by an increase in mitochondrial size and higher levels of mitochondrial protein complexes [134,135]. Oxidative phosphorylation then becomes prominent in mosquito stages, emerging as a key targetable process in sporogonic parasites [136,137,138,139,140]. The antimalarial drug atovaquone (**56**) inhibits cytochrome *bc1*, the third sub-complex of the ETC that acts as an electron acceptor, recycling ubiquinol to ubiquinone. The phenotypic effects of atovaquone in the ABS start to show slowly since the drug indirectly kills the parasites by depriving them of essential metabolites for DNA replication [141]. Atovaquone is active against gametocytes, although with IC_50_ values in the micromolar range [IC_50_(stage II–III) = 24 μM [142]; IC_50_(stage IV–V) = 5.31 [143], 16.1 [144], or 50.4 μM [142], depending on the study]. The activity of atovaquone is exerted during male gametogenesis (79% inhibition of male gamete exflagellation at 1 μM) and across ookinete development in the mosquito midgut, marking the essentiality of mitochondrial respiration at these stages [38,44,145]. Atovaquone significantly impaired sporogonic development and reduced the number of oocysts in the mosquito midgut following an indirect SMFA with an IC_50_ value of 2 nM [62]. Moreover, in luminescence-based SMFAs, atovaquone displayed > 99% inhibition at 5 μM in both the indirect and direct formats [62]. Strikingly, complete blockage of oocyst formation was observed by Paton et al. [145] in their new transmission-blocking method, which exploited atovaquone-coated surfaces for mosquito drug uptake (Figure 14). Atovaquone (100 μmol/m^2^) alone could completely block parasite transmission just after 6 min of exposure. Such a method overcomes the induction of any mechanism of vector resistance, since mosquito fitness is not affected, and the difficulties related to the indirect delivery of drugs into mosquitoes. Encouragingly, recent findings proved that this strategy is also as efficient against insecticide-resistant *Anopheles* mosquitoes and against *P. falciparum* resistant to front-line antimalarials (e.g., *Pf*Kelch13 resistant to artemisinins) [20]. Yet, using an approved antimalarial drug to reduce mosquito vectorial capacity and block transmission could further increase the risk of developing resistance to antimalarial drugs and compromising their efficacy in humans [146,147]. Thus, the use of new transmission-blocking compounds targeting the sporogonic stages of the parasites with novel modes of action and a deeper understanding of post-transmission biology will assist in the dissemination of this novel approach.

## 4. Conclusions

Due to the rapid development and ongoing propagation of resistance to first-line antimalarial drugs and insecticides, there is an urgent need for novel antimalarial therapeutics that target the spread of the disease. In this regard, transmission-blocking agents are the best option, as they not only interrupt the parasite’s life cycle but also prevent drug resistance. Considering the problems associated with the use of primaquine in G6PD-deficient individuals, scientists have been devising new transmission-blocking agents and repositioning non-malaria medications.

In this review, we have summarized a diverse subset of malaria transmission-blocking inhibitors by reporting compounds that have reached clinical stages, as well as those that have been described within the last three years. These compounds exhibit a wide range of modes of action, including altering *P. falciparum* epigenetic pathways, inhibiting kinases, or acting as antimetabolites. They represent important starting points for further development, with the aim of yielding clinical-stage inhibitors. Several reported compounds have both anti-asexual and transmission-blocking activities, which would be desirable in an antimalarial drug. However, the balance between the two activities is a significant issue for this class of compounds. In many instances, the anti-asexual action of the inhibitor is greater than its transmission-blocking function. As such, one of the goals of the scientific community would be to develop treatments with equivalent anti-asexual and transmission-blocking properties.

A further understanding of the regulatory mechanisms governing the maturation of gametocytes into differentiated sexual stages is pivotal to identifying new targets. This is of tremendous relevance, as there are numerous drugs demonstrated to serve as transmission blockers that have undiscovered targets, as evidenced by the many examples offered in this review. Indeed, understanding the molecular target will be crucial to allow combinations of transmission blockers with drugs active against the ABS, and this strategy might help prevent the emergence of resistance.

Several assays are currently available for evaluating the gametocytocidal activity of compounds [10,29,30,31,33,34,35,36,37], and the recent development of *Pf*DGFA in a high-throughput format has enabled the identification of compounds active specifically during gamete formation [24,38,40]. However, the identification of new chemotherapeutic interventions against specific parasite post-fertilization stages (i.e., zygote, ookinete, oocyst, and sporozoite development) is currently limited to low-throughput SMFAs [41,42,43] or *P. berghei*-based in vitro screens [18,19,148]. Targeting malaria parasites within mosquitoes is gaining traction in malaria eradication, as it is considered an effective strategy for preventing drug resistance and would reduce the antimalarial drug burden on humans [149]. Therefore, new robust in vitro systems that prioritize drug assessment and are amenable to high throughput are of paramount importance for accelerating the deployment of novel compounds inside mosquitoes. Remarkably, the recent discovery by Paton et al. [145] has added a novel approach to the malaria transmission-blocking arsenal, demonstrating that sporogony-targeting compounds such as atovaquone can be delivered to mosquito vectors through tarsal contact (e.g., via bed nets, indoor residual spraying), rendering them refractory to infection with no fitness cost. This method has the potential to reduce transmission even in endemic regions with a high prevalence of insecticide resistance, as it is equally effective on both pesticide-resistant and sensitive mosquitoes [145,149]. Nevertheless, knowledge gaps remain regarding targeting parasites inside mosquitoes. For instance, environment–host interactions, drug metabolism within mosquitoes, and potential new parasite resistance factors should be investigated further. While approaches like this are going forward, the development of new drug-testing tools is essential for accelerating drug discovery to identify additional compounds with potent antiparasitic activity during the mosquito stages of *P. falciparum* and enable the identification of novel targets that have been overlooked so far.

## Figures and Tables

**Table 1:** Epigenetic and kinase inhibitors showing multistage and transmission-blocking antiplasmodial activity against *P. falciparum* NF54.

Compound	Structure	Target Class	Asexual IC_50_ ± SEM (nM)	Stage I–II Gametocyte IC_50_ ± SEM (nM)	Stage IV–V Gametocyte IC_50_ ± SEM (nM)
SGI-1027 **8**	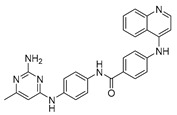	DNMT	50.8 ± 0.6	18.2 ± 2.1	322.4 ± 123.7
Chaetocin **9**	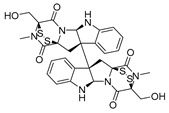	HMT	775.3 ± 366.0	292.3 ± 25.7	504.5 ± 92.3
BIX01294 **10a**	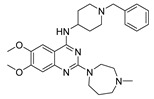	HMT	10.5 ± 3.6	12.3 ± 1.3	939.0 ± 84.7
UNC0631 **10b**	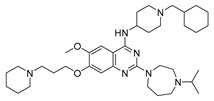	HMT	28.5 ± 5.9	14.8 ± 0.9	641.2 ± 83.0
UNC0642 **10c**	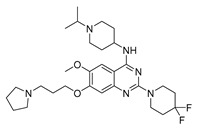	HMT	19.2 ± 10.4	14.6 ± 0.8	929.6 ± 199.0
UNC0379 **10d**	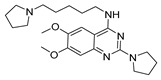	HMT	50.4 ± 2.3	21.3 ± 4.8	>1000
UNC0638 **10e**	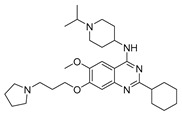	HMT	21.6 ± 2.0	16.4 ± 1.0	>1000
UNC0646 **10f**	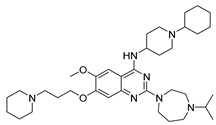	HMT	140.1 ± 3.8	66.8 ± 22.6	>1000
JIB-04 **11a**	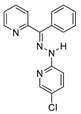	KDM	470.5 ± 28.3	133.1 ± 18.5	262.5 ± 113.0
Quisinostat **12a**	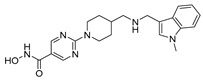	HDAC	<13	<13	148.1 ± 145.8
Panobinostat **13**	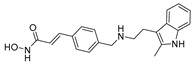	HDAC	8.7 ± 3.8	12.0 ± 4.8	515.3 ± 144.7
Apicidin **14**	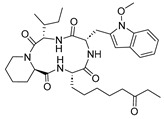	HDAC	23.1 ± 15.2	103.6 ± 2.9	590.2 ± 146.6
HC Toxin **15**	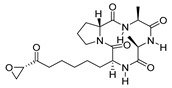	HDAC	15.1 ± 3.7	30.2 ± 0.1	351.4 ± 221.3
CUDC-101 **16**	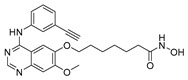	HDAC	35.6 ± 8.4	133.1 ± 6.3	2150.4 ± 744.3
Trichostatin A **17**	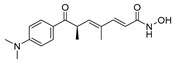	HDAC	62.3 ± 21.1	53.9 ± 5.4	3795.5 ± 1576.3
Dacinostat **18**	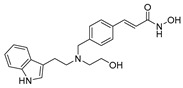	HDAC	40.8 ± 19.1	45.3 ± 0.9	2266.1 ± 843.2
Fedratinib **19**	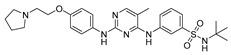	Kinase	66.9 ± 2.8	96.9 ± 19.7	>1000

## Data Availability

No new data were created or analyzed in this study.

## Abbreviations

ABSs	Asexual blood stages
Ac-COA	Acetyl coenzyme A
ACS	Acyl-CoA synthetase
AMS	Adenosine-5′-sulfamate
aaRSs	Aminoacyl-tRNA synthetases
ATP	Adenosine triphosphate
BCKDH	Branched-chain keto-dehydrogenase
CETSA	Cellular thermal shift assay
CYP	Cytochrome P450
DGFA	Dual gamete formation assay
DMSO	Dimethyl sulfoxide
DNMT	DNA methyltransferase
EC_50_	50% effective concentration
ED_90_	90% effective dose
EIA	Exflagellation inhibition assay
EM	Electron microscopy
ETC	Electron transport chain
FGAA	Female gamete activation assay
G6PD	Glucose-6-phosphate dehydrogenase
GAPDH	Glyceraldehyde-3-phosphate dehydrogenase
GFP	Green fluorescent protein
GST	Glutathione S-transferase
HDAC	Histone deacetylase
HEA	Hydroxyethylamine
HMT	Histone methyltransferase
IAP	Inhibitor of apoptosis
IC_50_	50% inhibitory concentration
IF	Immunofluorescence
iPanAms	Inverted pantothenamides
JmjC	Jumonji C
KDM	Lysine demethylase
LDH	Lactate dehydrogenase
MMV	Medicines for Malaria Venture
ODA	Ookinete development assay
PABP1	Polyadenylate-binding protein 1 complex
PCNA1	Proliferating cell nuclear antigen
*Pf*CLK3	*P. falciparum* cyclin-dependent-like kinase
*Pf*DHFR	*P. falciparum* dihydrofolate reductase
*Pf*ATP4	*P. falciparum* Na^+^-efflux ATPase ATP4
*Pf*PANK1	*P. falciparum* pantothenate kinase 1
*Pf*PI4Kβ	*P. falciparum* phosphatidylinositol 4-kinase type III β
*Pf*eEF2	*P. falciparum* translation elongation factor 2
*Pf*YRS	*P. falciparum* tyrosine-tRNA synthetase
*Pf*vapA	*P. falciparum* V-type H + -ATPase
Pf20S	*P. falciparum* 20S proteasome
PheRS	Phenylalanyl-tRNA synthetase
PK	Pharmacokinetics
PM	Plasmepsins
PMIX	Plasmepsin IX
PMX	Plasmepsin X
PVM	Parasitophorous vacuole membrane
RBCs	Red blood cells
SAHA	Suberoylanilide hydroxamic acid
SaLSSA	Saponin-lysis sexual stage assay
SAR	Structure–activity relationship
SMFA	Standard membrane feeding assay
SPR	Surface plasmon resonance
TAP	Triaminopyrimidine
YRS	Tyrosine-tRNA synthetase
WHO	World Health Organization
